# Can artificial intelligence applications improve food supply capacity? Evidence from 296 Chinese cities

**DOI:** 10.3389/fnut.2026.1877178

**Published:** 2026-07-01

**Authors:** Fangyan Bai, Chunyan Li, Qi Ban, Guodaohou Song

**Affiliations:** 1 College of Humanities and Social Development, Northwest A&F University, Xianyang, China; 2 School of China-ASEAN Institute of Statistics, Guangxi University of Finance and Economics, Nanning, China; 3 Postdoctoral Programme of Guangxi Beibu Gulf Bank, Nanning, China

**Keywords:** agricultural production efficiency, agricultural resource allocation efficiency, artificial intelligence, food supply capacity, per capita grain output

## Abstract

**Introduction:**

Against the backdrop of the rapid development of the digital economy, artificial intelligence (AI) has become an increasingly important driver of agricultural modernization and food supply capacity improvement. This study examines the impact of AI development on food supply capacity and further explores the mechanisms and regional differences underlying this relationship.

**Methods:**

Using panel data from 296 Chinese cities from 2010 to 2024, this study constructs a two-way fixed-effects model to evaluate the effect of AI development on food supply capacity. A series of robustness tests, endogeneity tests, lagged-effect tests, mechanism analyses, and heterogeneity analyses are conducted to verify the reliability of the findings and identify the channels through which AI exerts its influence.

**Results:**

The empirical results show that AI development significantly enhances food supply capacity, and this conclusion remains robust after multiple robustness and endogeneity checks. The lagged-effect analysis indicates that the positive impact of AI persists over time rather than being limited to the short term. Mechanism tests further reveal that AI improves food supply capacity mainly by increasing agricultural production efficiency and promoting the more efficient allocation of agricultural resources. Heterogeneity analysis shows that the promoting effect of AI is more pronounced in central China, major grain-producing regions, areas with relatively abundant precipitation, and regions with more advanced digital infrastructure.

**Discussion:**

These findings suggest that AI has gradually become a strategic technological support for agricultural modernization and the governance of national food supply capacity. Accordingly, policymakers should accelerate the construction of rural digital infrastructure, promote the integration of AI technologies into agricultural production systems, and formulate differentiated regional policies based on local agricultural foundations, resource endowments, and climatic conditions.

## 1 Introduction

Food security is a fundamental strategic issue closely linked to national economic development, social stability, and national security. With rapid global population growth, intensifying shifts in climate patterns, increasing constraints on environmental and resource-related conditions, and rising uncertainty in international grain markets, ensuring a stable food supply has become a major concern for countries worldwide (1). The UN Sustainable Development Agenda for 2030 explicitly proposes the goal of “Zero Hunger,” emphasizing the importance of improving agricultural productivity and strengthening the resilience of food systems. For China, food security has consistently remained a top national priority. The report delivered at the 20th National Congress of the Communist Party of China emphasized the importance of reinforcing food security through comprehensive and multidimensional approaches. Against the backdrop of labor aging in rural areas, increasing constraints on arable land resources, and rising agricultural production costs under traditional farming models, promoting the deep integration of next-generation information technologies with agriculture has become an essential pathway toward safeguarding food security (2).

This study is directly aligned with the research theme of artificial intelligence-enabled agri-food systems and food security. Food supply capacity represents the most fundamental availability dimension of the food security system, while artificial intelligence is reshaping grain production and supply systems through precision agriculture, smart agricultural machinery, remote sensing monitoring, pest and disease identification, intelligent irrigation, meteorological early warning, and supply chain coordination. Unlike general studies on the digital economy, artificial intelligence is characterized by intelligent recognition, predictive analytics, and automated decision-making, enabling it to more directly influence agricultural production decisions, factor input allocation, and production risk governance. Therefore, examining the impact of artificial intelligence development on food supply capacity not only helps deepen the understanding of the role of next-generation digital technologies in agricultural modernization, but also provides empirical evidence on how artificial intelligence can contribute to food security objectives.

In recent years, artificial intelligence (AI), supported by modern digital technologies such as large-scale data analytics, cloud computing architectures, Internet of Things (IoT) communication systems, and machine learning, has developed rapidly and has gradually penetrated multiple aspects of agricultural production, grain circulation, and agricultural governance (3). AI can improve agricultural productivity through applications such as remote sensing monitoring, pest and disease identification, precision fertilization, intelligent irrigation, and smart agricultural machinery scheduling (4). Moreover, AI contributes to enhancing the stability of grain production and strengthening agricultural resilience against risks through meteorological forecasting, supply chain coordination, and early-warning systems. Particularly in the context of the rapid development of digital agriculture and smart agriculture, AI is driving agricultural production from experience-based management toward data-driven and intelligent decision-making (5), thereby providing new technological support for food supply capacity. Consequently, systematically examining the impact of AI on food supply capacity and the transmission mechanisms behind this relationship has important practical implications for advancing agricultural modernization and improving national food supply capacity (6).

Existing studies have mainly investigated food supply capacity from the perspectives of the digital economy (7), agricultural technological advancement (8), agricultural mechanization (9), and agricultural modernization (10). Ye (11) argues that the growth of the digital economy and information technologies can enhance food supply capacity by improving agricultural productivity, optimizing resource allocation efficiency, and accelerating the diffusion of agricultural innovations. Lu and Huan (12) focus on the effects of agricultural technological innovation, agricultural mechanization, and fiscal support for agriculture on grain output. Nevertheless, the current literature still has several limitations. First, most studies primarily examine the overall effects of the digital economy or agricultural modernization on food supply capacity, while independent research specifically addressing AI as an emerging technology remains relatively limited. As a result, the specific mechanisms through which AI influences food supply capacity have not yet been sufficiently explored. Second, existing research mainly focuses on provincial-level analyses and lacks empirical evidence based on city-level panel data, making it difficult to accurately capture regional heterogeneity in AI development and food supply capacity. Third, the literature provides insufficient discussion of the transmission mechanisms through which AI affects food supply capacity, particularly with respect to agricultural production efficiency and agricultural resource allocation efficiency. Fourth, whether the impact of AI on food supply capacity varies across regions with different climatic conditions, grain production functions, and levels of digital infrastructure remains underexplored.

Based on this, this study uses panel data from 296 Chinese cities over the period 2010–2024 to systematically examine the impact of artificial intelligence development on food supply capacity, its underlying mechanisms, and regional heterogeneity. Compared with existing studies, the main contribution of this study lies in its direct examination of the relationship between artificial intelligence applications and food supply capacity. First, this study incorporates artificial intelligence development into the analytical framework of food security and explicitly examines its impact on food supply capacity, which represents a fundamental dimension of food security. In doing so, it extends the empirical evidence on artificial intelligence-enabled agri-food systems. Second, this study not only investigates whether artificial intelligence can improve food supply capacity, but also identifies the underlying mechanisms from the perspectives of agricultural production efficiency and agricultural resource allocation efficiency. This helps explain how artificial intelligence technologies can be transformed into enhanced grain production capacity. Third, using panel data from 296 Chinese cities between 2010 and 2024, this study provides city-level empirical evidence that enables a more refined identification of heterogeneous effects across regions with different levels of artificial intelligence development, agricultural production foundations, and digital infrastructure conditions. Fourth, this study further conducts heterogeneity analyses from the perspectives of regional differences, major grain-producing areas, precipitation conditions, and digital infrastructure levels. These analyses provide empirical evidence for understanding the applicable conditions and policy boundaries of artificial intelligence in enhancing food supply capacity.

It should be noted that food security is a multidimensional concept, generally encompassing availability, access, utilization, and stability. Due to the limitations of continuous and comparable city-level data, this study does not equate per capita grain output with food security in its full sense. Instead, per capita grain output is defined as a proxy for “food supply capacity,” and the analysis focuses on the effect of artificial intelligence on the availability dimension of food security. Therefore, the findings of this study are mainly applicable to food supply capacity and cannot fully represent the impact of artificial intelligence on food access, nutritional utilization, or long-term supply stability.

## 2 Literature review

### 2.1 Studies on the determinants of food supply capacity

Food security serves as an important basis for national economic security and social stability and has long been a core issue in the field of agricultural economics (13). Based on the definition provided by the United Nations Food and Agriculture Organization (FAO), food security encompasses not only the adequacy of food supply, but also food accessibility, food utilization efficiency, and the stability of food supply systems. With rapid population growth, tightening resource and environmental constraints, and increasing volatility in international grain markets, the focus of food security research has gradually shifted from merely emphasizing grain output toward broader concerns regarding agricultural productivity, agricultural sustainability, and the resilience of agricultural systems (14).

Gewa et al. (15) examined food supply capacity from the perspective of traditional agricultural production factors, arguing that cultivated land, labor, capital investment, and agricultural infrastructure are key determinants of grain production capacity. Deng et al. (16) found that agricultural capital investment and fiscal support for agriculture can significantly enhance grain production capacity and improve the stability of agricultural production. Meanwhile, improvements in agricultural mechanization can alleviate labor shortages in rural areas, reduce production costs, and increase agricultural labor productivity, thereby strengthening food supply capacity (55).

With the continuous advancement of agricultural modernization, agricultural technological progress has gradually become a major focus of food supply capacity research (17). Mozumdar (18) suggests that agricultural technological innovation can promote food supply capacity by improving agricultural total factor productivity, enhancing resource utilization efficiency, and strengthening agricultural resilience against risks. Particularly under the context of climate change, technological progress in agriculture plays a critical role in stabilizing grain production (19). In addition, climatic conditions are widely recognized as an important determinant of food supply capacity (20). Changes in precipitation (21), extreme weather events (22), and natural disasters (23) can significantly affect the stability of agricultural production and further influence food supply capacity. Consequently, enhancing the adaptive capacity of agricultural systems through technological progress has become an important topic in food supply capacity research.

Overall, existing studies on the determinants of food supply capacity have established a relatively comprehensive theoretical foundation. However, most research still focuses primarily on traditional agricultural production factors and agricultural technological progress, while insufficient attention has been paid to how emerging digital technologies such as artificial intelligence influence food supply capacity.

### 2.2 Studies on artificial intelligence and agricultural development

Along with the rapid expansion of the digital economy, the integration of digital technologies with agriculture has gradually become a major trend in agricultural modernization (24). Liang and Qiao (25) argue that the digital economy can promote high-quality agricultural development by improving information transmission efficiency, reducing transaction costs, facilitating the diffusion of agricultural technologies, and optimizing agricultural resource allocation. In particular, the widespread application of digital technologies such as the Internet, big data, cloud computing, and the Internet of Things (IoT) has provided important support for the intelligent and precision-oriented transformation of agricultural production (26).

In recent years, artificial intelligence, as a key element of the digital economy, has been increasingly applied in the agricultural sector. Relying on technologies including intelligent algorithms, neural network technologies, visual recognition systems, and large-scale data analysis, AI enables intelligent identification, predictive analysis, and automated control throughout the agricultural production process (27). Aziz et al. (28) indicate that AI has been widely used in precision agriculture, pest and disease detection, agricultural remote sensing, meteorological forecasting, intelligent irrigation, and smart agricultural machinery, significantly improving agricultural productivity and production stability.

On the one hand, AI can enhance agricultural production efficiency (29). Through agricultural data analysis and intelligent decision-making, AI improves precision management during the production process and reduces resource waste and inefficient input utilization (30). For example, intelligent irrigation systems can automatically adjust irrigation intensity according to soil moisture and meteorological conditions, while pest and disease identification systems can promptly detect agricultural risks and reduce production losses, thereby increasing agricultural output per unit of input. On the other hand, AI can also strengthen agricultural risk governance capacity (31). Through applications such as agricultural weather forecasting, natural disaster monitoring, and agricultural supply chain coordination, AI enhances the stability and resilience of agricultural systems in response to external shocks (32).

Although existing studies have begun to explore the application of AI in agriculture, research specifically examining the relationship between AI and food supply capacity remains relatively limited. Most existing studies focus on the digital economy as a whole, smart agriculture, or agricultural modernization, while systematic analyses of the independent role of AI are still scarce. Moreover, current research pays greater attention to agricultural output, green agricultural development, and agricultural efficiency, whereas discussions regarding food supply capacity as a comprehensive objective remain insufficient. In addition, there is still a lack of in-depth analysis concerning the specific pathways through which AI influences food supply capacity and the regional differences in these effects.

It should be noted that the role of artificial intelligence in the agri-food system is not limited to agricultural production. With the deepening application of digital technologies across the agri-food supply chain, AI can also affect food security through food processing, storage management, cold-chain logistics, distribution, quality traceability, and market demand forecasting. A recent systematic review of AI and the agri-food supply chain indicates that AI can enhance decision-making capabilities and transparency within the agri-food supply chain and promote the development of personalized nutrition strategies. At the same time, AI adoption also faces barriers related to technological accessibility, knowledge gaps among stakeholders, and consumer skepticism. Therefore, the impact of AI on food security is clearly systemic, extending beyond the production stage to post-harvest management, supply chain coordination, and consumer acceptance.

### 2.3 Studies on agricultural production efficiency and resource allocation efficiency

Agricultural production efficiency and agricultural resource allocation efficiency are important determinants of food supply capacity and key indicators of high-quality agricultural development (33). Agricultural production efficiency mainly reflects the relationship between inputs and outputs in agricultural production and essentially represents the combined effects of technological progress and management capability in agriculture. Wang (34) agree that improving agricultural production efficiency can effectively strengthen food supply capacity and promote sustainable agricultural development.

In studies of agricultural production efficiency, agricultural total factor productivity is commonly regarded as an important indicator for measuring agricultural efficiency (35). Po-Chi et al. (36) have found that agricultural technological progress, improvements in agricultural mechanization, and enhancements in agricultural infrastructure can significantly increase agricultural TFP. Over the past few years, with the widespread application of digital technologies, an increasing number of studies have begun to investigate the impact of the digital economy and digital technologies on agricultural production efficiency. Deichmann et al. (37) suggests that digital technologies can improve agricultural efficiency through information sharing, precision management, and intelligent control, thereby promoting the shift in agricultural production from extensive growth to intensive development.

Meanwhile, agricultural resource allocation efficiency has also become an important research focus (37). Traditional agricultural production often suffers from inefficient allocation of land, labor, capital, and agricultural production materials, resulting in resource waste and declining agricultural productivity (38). Chang (39) argue that digital technologies and informatization can alleviate resource misallocation by improving information transparency, reducing information asymmetry, and facilitating the rational movement of agricultural production resources. AI further strengthens the optimization of agricultural resource allocation. Through intelligent decision-making and data analysis, AI enables precision input allocation and improves the utilization efficiency of production factors such as land, machinery, fertilizers, and agricultural electricity (40). Therefore, efficiency of agricultural production and agricultural resource allocation efficiency may constitute important transmission channels through which AI affects food supply capacity. However, systematic empirical examination of these mechanisms remains insufficient in the existing literature.

### 2.4 Literature review summary and research gap

In summary, existing studies have examined food supply capacity from multiple perspectives, including agricultural production factors, agricultural technological progress, the digital economy, and agricultural modernization, thereby providing an important theoretical foundation for this study. Nevertheless, several limitations remain.

First, previous research mainly concentrates on the overall impact of the digital economy or agricultural modernization on food supply capacity, while independent research on AI as an emerging digital technology remains relatively limited. Unlike traditional digital technologies, AI has stronger technological characteristics in intelligent identification, predictive analysis, and automated control, making it necessary to separately investigate its impact on food supply capacity. Second, most previous studies rely mainly on provincial-level data, with insufficient empirical evidence based on city-level analyses. Since substantial differences exist across Chinese cities in agricultural endowments, digital infrastructure, and AI development levels, provincial-level data may fail to adequately capture regional heterogeneity. Therefore, research based on city-level panel data has stronger practical relevance. Third, existing research provides insufficient discussion of the mechanisms through which AI affects food supply capacity. Most studies remain at the level of correlation analysis and lack systematic examinations of mediating mechanisms such as agricultural production efficiency and agricultural resource allocation efficiency, thereby failing to fully reveal the internal logic through which AI empowers food supply capacity. Fourth, current studies pay relatively limited attention to regional and climatic heterogeneity. Considerable regional disparities can be observed in relation to grain production functions, digital infrastructure, precipitation conditions, and agricultural modernization levels. Consequently, the impact of AI on food supply capacity may vary substantially across regions.

Based on these considerations, this study utilizes panel data covering 296 Chinese cities from 2010 to 2024 to systematically investigate the impact of AI development on food supply capacity, its underlying mechanisms, and regional heterogeneity. The study aims to provide new theoretical insights and empirical evidence for promoting agricultural modernization and safeguarding national food security through AI empowerment.

## 3 Theoretical analysis and research hypotheses

### 3.1 Artificial intelligence and food supply capacity

The effect of artificial intelligence on food supply capacity essentially reflects the way in which a new generation of digital technologies reshapes agricultural production decision-making, factor allocation, and risk governance (41). Unlike traditional agricultural production, which relies largely on experiential judgment, AI can enhance information-processing capacity in agricultural production through data collection, algorithmic analysis, and intelligent decision-making, thereby reducing information incompleteness and decision-making uncertainty. For grain production, producers’ timely access to information on soil conditions, weather, pests and diseases, markets, and crop growth directly affects the quality of decisions in key stages such as sowing, fertilization, irrigation, harvesting, storage, and transportation. Therefore, AI development helps improve the scientific and precision-oriented management of grain production (42).

From the perspective of the production process, AI can strengthen food supply capacity by improving the efficiency of agricultural production decision-making and production process control (43). On the one hand, AI can improve the input–output matching relationship in agricultural production by enabling more precise allocation of production factors such as water, fertilizer, pesticides, machinery, and labor to grain production activities, thereby reducing ineffective inputs and resource waste. On the other hand, AI facilitates the transition of agricultural production from extensive management to precision-oriented and intelligent management, thereby improving output per unit of resources and grain production efficiency.

From the perspective of risk governance, grain production is highly dependent on natural conditions and is vulnerable to extreme weather events, pests and diseases, natural disasters, and market fluctuations (32). AI can improve the identification of agricultural production risks through predictive analytics, dynamic monitoring, and early-warning feedback, enabling producers to adjust production arrangements more promptly and reduce potential losses. Therefore, AI may not only improve grain production efficiency but also enhance the stability of grain production systems in response to external shocks (44).

In summary, AI development can improve food supply capacity by enhancing agricultural information-processing capacity, increasing production decision-making efficiency, optimizing the allocation of production factors, and strengthening risk governance (27). Based on this analysis, this study proposes the following research hypothesis:

*H1*: AI development significantly improves food supply capacity.

### 3.2 The mediating role of agricultural production efficiency

Agricultural production efficiency constitutes an important foundation of food supply capacity and is also a key indicator of agricultural modernization (45). Improvements in agricultural production efficiency imply that higher grain output can be achieved under given resource input conditions, thereby enhancing food supply capacity. AI can improve agricultural production efficiency through intelligent identification, automated control, and precision management (46).

Specifically, AI can improve agricultural technical efficiency (47). Through agricultural data analysis and intelligent decision-making, AI can optimize cropping structures, input intensity, and agricultural machinery scheduling during the production process, thereby improving the precision and efficiency of agricultural management. Meanwhile, AI can reduce information asymmetry and management costs in agricultural production, thereby minimizing inefficient input use and resource waste (48). In addition, AI can enhance agricultural labor productivity and agricultural total factor productivity. Against the backdrop of declining agricultural labor supply and rural labor aging, smart agricultural machinery and automated equipment can effectively replace part of the traditional agricultural labor force, thus improving agricultural productivity and grain production capacity. Accordingly, agricultural production efficiency may constitute an important mediating mechanism through which AI affects food supply capacity. Drawing upon the above theoretical analysis, this study proposes the following hypothesis:

*H2*: Artificial intelligence promotes food supply capacity by improving agricultural production efficiency.

### 3.3 The mediating role of agricultural resource allocation efficiency

Agricultural resource allocation efficiency reflects the rationality of allocating production factors across different agricultural production processes and serves as an important determinant of grain production capacity (49). Traditional agricultural production is often characterized by inefficient allocation of land, labor, capital, and agricultural production materials, which can lead to resource waste and lower agricultural productivity. AI can improve agricultural resource allocation efficiency through data analytics and intelligent decision-making (50).

On the one hand, AI can enhance information transparency in agriculture (51). Through agricultural data platforms and intelligent monitoring systems, agricultural producers can obtain more accurate information regarding agricultural markets, climatic conditions, and production activities, thereby optimizing production decisions and reducing resource misallocation (52). On the other hand, AI enables precision input allocation in agricultural production (53). For example, precision fertilization and intelligent irrigation systems can adjust input intensity according to actual production needs, thereby improving the utilization efficiency of agricultural production materials.

Moreover, AI can improve the mobility and allocation efficiency of agricultural production factors (29). Through digital agricultural platforms and supply chain coordination, production factors such as agricultural labor, capital, and machinery can flow more efficiently toward high-productivity sectors, thereby improving overall efficiency of agricultural resource allocation. Therefore, the efficient allocation of agricultural resources may serve as an important transmission channel through which AI influences food supply capacity. Drawing upon the above theoretical analysis, this study proposes the following hypothesis:

*H3*: Artificial intelligence promotes food supply capacity by improving agricultural resource allocation efficiency.

## 4 Research design

### 4.1 Variable selection

#### 4.1.1 Dependent variable

*Food supply capacity (FSC)*: This study uses per capita grain output at the prefecture-level city scale as the proxy variable for food supply capacity. It should be noted that “total grain output” in this study does not refer only to wheat output. Rather, it is a comprehensive grain production indicator under China’s official statistical classification and generally includes rice, wheat, corn, and other grain crops. This indicator reflects the per capita grain production level of a given region during a specific period and mainly corresponds to the availability dimension of the FAO food security framework. Since food security also includes access, utilization, and stability dimensions, this study explicitly defines the dependent variable as food supply capacity rather than equating it with food security in its full sense. To ensure the robustness of the results, total grain output at the city level is further used as an alternative dependent variable in the robustness test.

#### 4.1.2 Explanatory variable

*Artificial intelligence development level (AI)*: This study constructs a city-level AI development indicator system from three dimensions: infrastructure construction, enterprise production and application, and government support. Infrastructure construction mainly reflects the computing-power foundation and network connectivity conditions required for AI development, including AI computing power and Internet penetration. Enterprise production and application mainly captures the agglomeration, innovation, and application of AI technologies within the urban industrial system, including the number of AI firms, the number of AI patents, and industrial robot penetration. Government support mainly reflects local governments’ policy attention and fiscal investment in AI development, including the proportion of AI-related policy term frequency and fiscal expenditure on science and technology.

Government support in terms of policy guidance and fiscal investment in science and technology plays an important role in promoting the development of the AI industry. By issuing AI-related policies, increasing fiscal expenditure on science and technology, and improving the innovation environment, local governments provide institutional and resource support for AI technology research and development, industrial agglomeration, and application diffusion. Based on the availability of city-level data, this study measures government support mainly from two aspects: government policy attention and fiscal investment in science and technology. These are measured by the proportion of AI-related policy term frequency and the share of science and technology expenditure in total government fiscal expenditure, respectively. Accordingly, this study constructs a city-level AI development indicator system, as shown in Table 1, to more systematically reflect the development of regional AI technological innovation and application.

**Table 1:** Measurement of artificial intelligence development level.

Variable	Dimension	Sub-dimension	Indicator	Measurement	Data source
AI development level	Infrastructure construction	Hardware foundation	AI computing power	Number of graphics card imports	China Customs Database
		Connectivity capacity	Internet penetration	Number of internet users	China City Statistical Yearbook
	Enterprise production and application	Enterprise agglomeration	Number of AI enterprises	Number of artificial intelligence enterprises	Qichacha enterprise credit information platform; obtained by Python web scraping
		Technological innovation capacity	Number of AI patents	Number of granted artificial intelligence patents	China National Intellectual Property Administration
		Depth of enterprise application	Robot adoption	Industrial robot penetration rate	International Federation of Robotics (IFR)
	Government support	Government attention	Policy intensity	Share of AI-related policy word frequency	Local government websites; obtained by Python web scraping
		Government investment	Fiscal science and technology input	Share of science expenditure in government fiscal expenditure	China City Statistical Yearbook

The AI development index is constructed based on the seven specific indicators listed in the table. This study first applies positive-direction transformation and dimensionless normalization to each indicator. The entropy weight method is then used to determine the weight of each indicator, and the standardized indicators are aggregated through weighted summation to obtain the city-year-level AI development index. The dimensional classification in the table is mainly used for theoretical categorization. In the index aggregation process, the specific indicators are used as the weighting units, and no additional subjective weights are assigned to the dimensions.

It should be noted that this study uses the number of graphics card imports as a proxy for AI computing power. This is mainly because high-performance computing hardware, such as GPUs, provides an important foundation for AI model training, data processing, and the operation of intelligent algorithms, and can therefore reflect, to some extent, the computing-power support conditions for urban AI development. However, GPU imports cannot be fully equated with the actual deployment level of agricultural AI. On the one hand, GPUs may be used in many fields, such as industry, finance, transportation, and Internet services, and do not necessarily directly serve agricultural production. On the other hand, import data mainly reflect the basic AI capacity at the city level, but cannot identify the intensity of AI application in specific agricultural scenarios such as precision agriculture, smart agricultural machinery, agricultural remote sensing, and agrometeorological forecasting. Therefore, this study interprets GPU imports as a proxy for the basic conditions of urban AI development, rather than as a direct measure of the application level of agricultural AI.

Similarly, Internet penetration mainly reflects a city’s digital connectivity and information infrastructure. A higher Internet penetration rate facilitates data transmission, access to agricultural information, the operation of digital platforms, and the diffusion of AI technologies. However, it does not directly capture the extent to which agricultural AI is applied in scenarios such as planting, irrigation, agricultural machinery scheduling, or pest and disease identification. Therefore, Internet penetration is more appropriately regarded as a proxy for the AI development environment and digital infrastructure conditions, rather than as a direct measure of the depth of agricultural AI application.

To improve comparability across cities of different sizes, this study does not directly include the total number of AI patents or the total number of industrial robots in the indicator system when constructing the AI development index. Instead, scale-related indicators are standardized before being incorporated into the index. Specifically, the AI patent indicator is measured using city-size-adjusted AI patent intensity, which can be expressed as the number of AI patents per 10,000 people or the number of AI patents per unit of GDP. This measure captures the intensity of urban AI technological innovation rather than merely reflecting differences in city size. The industrial robot indicator is measured using industrial robot density or industrial robot penetration, defined as the ratio of industrial robot use to manufacturing employment or industrial employment, in order to reflect the intensity of robot application within the urban industrial system.

After completing the above scale standardization, this study further applies dimensionless normalization to AI patent intensity, industrial robot density, and other AI-related indicators. The entropy weight method is then used to calculate the weights of the indicators and construct the city-year-level AI development index. This procedure helps reduce the influence of differences in population size, economic scale, and industrial scale on the measurement results, thereby improving the comparability of AI development levels across cities of different sizes.

Ideally, the development level of agricultural AI should be measured using more agriculture-specific indicators, such as the number of agricultural AI firms, the number of smart agriculture patents, the density of smart agricultural machinery and equipment, the coverage rate of agricultural Internet of Things devices, the level of digital farmland construction, the number of smart agriculture demonstration zones, the extent of agricultural remote sensing monitoring applications, the coverage rate of intelligent irrigation systems, and the proportion of agricultural producers using digital platforms. These indicators would more directly reflect the actual application of AI technologies in agricultural production and food supply processes. However, due to limitations in the availability of long-term and continuous city-level data, these agriculture-specific indicators are currently difficult to construct as a complete panel dataset for 296 cities over the period 2010–2024. Therefore, this study uses a city-level composite AI development index as the core explanatory variable to capture the overall technological environment, infrastructure conditions, enterprise innovation capacity, and government support generated by AI development. Future research could construct a specialized agricultural AI index when data conditions improve, thereby identifying more precisely the impact of AI applications in agricultural scenarios on food supply capacity.

In terms of index aggregation, this study adopts the entropy weight method to construct the composite city-level AI development index. The entropy weight method is selected because it can objectively determine indicator weights based on the dispersion and information contribution of each indicator in the sample, thereby avoiding the potential subjectivity associated with equal weighting while preserving the economic meaning of each indicator. Specifically, this study first applies dimensionless normalization to all positive indicators listed in Table 1. Since the selected indicators, including AI computing power, Internet penetration, the number of AI firms, the number of AI patents, industrial robot penetration, the proportion of AI-related policy term frequency, and fiscal investment in science and technology, are all positive indicators, the range standardization method is used for processing.

#### 4.1.3 Mediating variables

##### 4.1.3.1 Agricultural production efficiency (APE)

This study further incorporates agricultural production efficiency as a mediating variable to examine the internal mechanism through which artificial intelligence (AI) influences food supply capacity. Existing studies suggest that AI technologies can improve agricultural production efficiency and promote agricultural total factor productivity through applications such as precision agriculture, intelligent identification, agricultural forecasting, and automated control. Therefore, agricultural production efficiency can effectively reflect the efficiency-enhancing mechanism of AI-enabled agricultural production.

Drawing upon the existing literature, this study uses agricultural total factor productivity (TFP) as the proxy indicator for agricultural production efficiency. Following the DEA-Malmquist index approach, agricultural labor input, agricultural machinery investment, fertilizer application, and agricultural electricity consumption are selected as input variables, while grain output is employed as the output variable for measurement.

##### 4.1.3.2 Agricultural resource allocation efficiency (ARE)

Relevant studies argue that AI can alleviate resource misallocation problems in agricultural production through data analytics, intelligent decision-making, and precision input allocation, thereby improving the allocation efficiency of production factors such as land, labor, capital, machinery, and agricultural production materials, ultimately enhancing grain production capacity.

According to prior research findings, this study adopts an agricultural resource allocation efficiency index to measure this mediating variable. Specifically, following the SBM-DEA model framework, agricultural labor input, crop-sown area, agricultural machinery investment, agricultural electricity consumption, and fertilizer application are selected as input indicators, while grain output is treated as the desirable output to construct the agricultural resource allocation efficiency index. Furthermore, to enhance the robustness of the indicator, supplementary tests are conducted using factor misallocation indices for land, capital, and labor.

#### 4.1.4 Control variables

To reduce possible estimation bias arising from omitted factors, this study follows Li et al. (2021) and controls for the following variables. Fiscal expenditure (FE) is measured by the ratio of local general public budget expenditure to regional gross domestic product (GDP), reflecting the level of government public investment. Agricultural population (PEO) is measured by the ratio of employees in the primary industry to the total regional population, capturing the scale of agricultural labor input. Value added of the primary industry (AVPI) is measured by the value added generated by the primary industry, representing the level of agricultural economic development. Gross domestic product (GDP) is measured as the natural logarithm of each city’s regional GDP and is used to control for differences in the overall level of regional economic development and economic scale. Per capita electricity consumption of agricultural machinery (MAP) is measured by the ratio of agricultural electricity consumption to the agricultural population, reflecting the level of agricultural mechanization and electricity input in agricultural production. Fertilizer application (FER) is measured by the amount of agricultural fertilizer used, representing the intensity of agricultural production input.

These variables collectively control for regional heterogeneity that may affect the dependent variable from the perspectives of fiscal support, labor input, industrial development, economic foundations, and agricultural factor inputs.

Summary statistics for the variables are presented in Table 2.

**Table 2:** Descriptive statistics of variables.

Variable	Obs	Mean	Std. dev.	Min	Max
fsc	4,421	0.462	0.500	0.0003	5.498
ai	4,421	6.031	1.866	1.0986	12.237
fer	4,421	0.269	0.221	0.0018	2.083
map	4,421	0.747	5.411	0.0029	303.746
gdp	4,421	16.401	2.227	13.855	20.106
peo	4,421	2.482	6.346	0.1	59.4
avpi	4,421	12.174	7.949	0.03	49.89
fe	4,421	0.206	0.128	0.0438	1.554

### 4.2 Model specification

#### 4.2.1 Baseline regression model

To empirically investigate the impact of artificial intelligence (AI) development on food supply capacity, this study employs a two-way fixed-effects model incorporating both individual fixed effects and time fixed effects. This specification effectively controls for unobservable individual heterogeneity that remains constant over time, as well as common temporal shocks affecting all sample units, thereby improving the accuracy of estimating the effect of AI development on food supply capacity. The baseline model is shown in Equation (1):

$$\begin{array}{l} {\mathrm{gsc}}_{i,t}=\beta_{1}{\mathrm{ai}}_{i,t}+\beta_{2}{\mathrm{gdp}}_{i,t}+\beta_{3}{\mathrm{fe}}_{i,t}+\beta_{4}{\mathrm{peo}}_{i,t}+\beta_{5}{\text{avpi}}_{i,t}\\ +\beta_{5}{\mathrm{map}}_{i,t}+\beta_{6}{\mathrm{fer}}_{i,t}+u_{i}+\varrho_{t}+\alpha +\varepsilon_{i,t} \end{array}$$ (1)

${\mathrm{gsc}}_{i,t}{\mathrm{ai}}_{i,t}u_{i}\varrho_{t}\varepsilon_{i,t}$ where FSC_*it* denotes the dependent variable, namely the level of food supply capacity in city *i* during year *t*; AI_*it* represents the core explanatory variable measuring the level of artificial intelligence development; and the remaining variables are control variables included in the model. *μ*_*i* denotes city fixed effects, which control for unobservable heterogeneous characteristics that remain constant over time across cities. *λ*_*t* represents year fixed effects, which capture time-specific factors that commonly affect all observations, such as policy adjustments and macroeconomic fluctuations. *α* is the constant term, and *ε*_*it* denotes the random error term, reflecting other unobservable random factors not explained by the model.

#### 4.2.2 Mediation effects model

To analyze the mediating pathways through which artificial intelligence (AI) development affects food supply capacity, this study constructs a mediation effect model to identify the potential transmission channels underlying this relationship. Drawing on the methodological framework proposed by (56), traditional single-mediator testing approaches may suffer from certain limitations in empirical applications, potentially leading to biased estimation results or insufficient statistical power in identifying mediation effects. Therefore, based on the classical mediation testing framework developed by Baron and Sobel, this study adopts a more comprehensive mediation effect testing strategy.

Compared with conventional methods, the adopted approach exhibits several advantages in terms of statistical performance. First, it helps improve the statistical power of the estimation results, thereby enhancing the model’s ability to identify mediation mechanisms. Second, it better controls for both Type I and Type II errors, reducing the probability of incorrectly accepting or rejecting the existence of mediation effects, and thus improving the accuracy and robustness of the empirical findings.

Accordingly, the following regression models, Equations 2 and 3, are specified to examine the specific mechanisms through which AI development influences food supply capacity. The models are shown in Equations (2) and (3):

$$\frac{{\mathrm{ape}}_{i,t}}{{\mathrm{are}}_{i,t}}=\beta_{1}{\mathrm{ai}}_{i,t}+\beta_{2}Z_{i,t}+u_{i}+\varrho_{t}+\alpha +\varepsilon_{i,t}$$ (2)

$${\mathrm{gsc}}_{i,t}=\beta_{1}{\mathrm{ai}}_{i,t}+\sigma_{1}\frac{\mathrm{ape}}{\mathrm{are}}+\beta_{2}Z_{i,t}+u_{i}+\varrho_{t}+\alpha +\varepsilon_{i,t}$$ (3)

${\mathrm{ape}}_{i,t},{\mathrm{are}}_{i,t}$ where APE_*it* and ARE_*it* denote agricultural production efficiency and agricultural resource allocation efficiency, respectively. The remaining variables are defined in the same manner as those used in the baseline model.

### 4.3 Data sources

Since China’s artificial intelligence (AI) industry entered a stage of rapid development around 2010, this study selects 296 Chinese cities during the period from 2010 to 2024 as the research sample in order to examine the impact of AI development on food supply capacity and its underlying mechanisms. To ensure the reliability and accuracy of the empirical results, variables with missing observations for three consecutive years or more were excluded. In addition, all core variables were winsorized at the upper and lower 1% levels to mitigate the influence of extreme values. After data cleaning and processing, an unbalanced panel dataset consisting of 4,421 city-year observations was obtained.

Regarding data sources, the AI computing power data used in constructing the AI development index were obtained from the China Customs Database. Data on the number of AI-related enterprises were collected from the Qichacha Enterprise Credit Information Platform. AI patent data were obtained from the China National Intellectual Property Administration, while industrial robot data were obtained from the International Federation of Robotics (IFR). The frequency of AI-related policy terms was manually compiled from local government official websites. Data for the dependent variable, control variables, and other macroeconomic and socioeconomic indicators were primarily collected from the China City Statistical Yearbook and annual statistical bulletins issued by local statistical bureaus.

## 5 Empirical results

### 5.1 Baseline regression results

Table 3 presents the benchmark regression results for the effect of AI development on food supply capacity. As shown in Column (1), when only city fixed effects and time fixed effects are controlled for, the estimated coefficient of AI development (ai) is 0.0516 and is statistically significant at the 1% level, indicating that AI development can significantly improve food supply capacity.

**Table 3:** Baseline regression results.

Variables	(1) fsc	(2) fsc
ai	0.0516*** (5.166)	0.0686*** (5.314)
fer		−0.0120 (−0.599)
map		−0.000767** (−2.020)
gdp		−0.154*** (−4.749)
peo		−0.00343** (−2.372)
avpi		0.00362 (1.394)
fe		−0.166** (−2.060)
Year fixed effects	Yes	Yes
City fixed effects	Yes	Yes
Constant	0.151** (2.500)	2.582*** (5.449)
Observations	4,421	4,421
*R*-squared	0.034	0.079
Number of cities	296	296

***, **, and * indicate statistical significance at the 1%, 5%, and 10% levels, respectively. Robust heteroskedasticity-consistent *t*-statistics clustered at the city level are reported in parentheses. The same notation applies hereafter.

After further incorporating control variables such as fiscal expenditure, agricultural labor input, regional economic development, agricultural machinery investment, and agricultural production factor inputs, the estimated coefficient of the AI variable in Column (2) increases to 0.0686 and remains significant at the 1% level. This finding suggests that even after controlling for unobservable heterogeneity across cities and other important factors potentially affecting food supply capacity, AI still exhibits a stable and significant promoting effect, thereby demonstrating the strong reliability of the empirical results.

From an economic perspective, AI can enhance food supply capacity through several channels, including improving agricultural production efficiency, optimizing agricultural resource allocation, and strengthening agricultural risk management capabilities. On the one hand, the application of AI technologies in precision agriculture can improve crop production efficiency, reduce production costs, and enhance the scientific management of agricultural production. On the other hand, AI can strengthen agricultural meteorological forecasting, pest and disease identification, and agricultural supply chain management, thereby reducing uncertainty risks in the agricultural production process. In addition, the development of AI contributes to the digital transformation of agriculture and improves the efficiency of agricultural technology diffusion, which further enhances the stability and resilience of grain production systems. Therefore, AI is not only an important technological force driving agricultural modernization, but also a critical support for safeguarding food supply capacity.

Regarding the control variables, different variables exhibit heterogeneous effects on food supply capacity. Specifically, the estimated coefficient of regional gross domestic product (GDP) is significantly negative, indicating that regions with higher levels of economic development may experience a certain degree of suppression in food supply capacity. One possible explanation is that economic growth is often accompanied by accelerated industrialization and urbanization, which may lead to the conversion of agricultural land for non-agricultural purposes and consequently weaken grain production capacity. Meanwhile, labor migration from agriculture to non-agricultural sectors in economically developed regions may reduce agricultural labor supply and generate a crowding-out effect on grain production.

In addition, both agricultural population (PEO) and fiscal expenditure (FE) exhibit significantly negative effects on food supply capacity. The negative effect of agricultural labor may indicate that simply increasing agricultural labor input does not necessarily improve food supply capacity, as the traditional labor-intensive agricultural model is no longer well suited to the efficiency requirements of modern agricultural development. The negative coefficient of fiscal expenditure may reflect that local fiscal resources are more heavily allocated to infrastructure construction, industrial support, and urban public services, while support for agriculture remains relatively insufficient.

Moreover, per capita electricity consumption of agricultural machinery (MAP) also shows a significantly negative effect, suggesting that agricultural machinery investment may have limited effectiveness in promoting food supply capacity if it lacks technological coordination and large-scale operational support. Overall, the marginal contribution of traditional factor inputs to food supply capacity is gradually declining, whereas the importance of technological progress—particularly AI technology—is continuously increasing.

It should be noted that the R-squared of the baseline model in Table 3 is 0.079, indicating that the model has relatively limited overall explanatory power for variations in urban food supply capacity. This result does not imply that the effect of the AI variable is unimportant. Rather, it reflects the complexity of food supply capacity and the fact that it is determined by multiple factors. The primary purpose of the baseline model in this study is to identify the marginal effect of AI development on food supply capacity, rather than to fully predict all variations in urban food supply capacity. In the two-way fixed-effects model, city fixed effects and year fixed effects absorb time-invariant city characteristics and common year-specific shocks. The remaining explanatory power mainly comes from time-varying factors within cities. Therefore, when the dependent variable is affected by numerous short-term natural shocks and region-specific factors, the model’s R-squared may be relatively low (54).

Specifically, several important factors may still affect urban food supply capacity but cannot be fully incorporated into the baseline model due to limitations in the availability of long-term continuous city-level data. First, natural resource and ecological conditions may still play an important role, including cultivated land quality, soil fertility, topographic conditions, water resource endowment, and irrigation infrastructure, all of which directly affect grain yield per unit area and production stability. Second, climatic and disaster shocks exhibit strong annual fluctuations. Extreme heat, floods, droughts, pests and diseases, and other natural disasters may all cause short-term fluctuations in grain output. Third, differences in agricultural management systems and farmers’ behavior may also affect food supply capacity, including the degree of land transfer, the level of large-scale farming, the coverage of agricultural socialized services, farmers’ willingness to plant grain crops, and the comparative returns of grain production. Fourth, local agricultural policies, fiscal subsidies, grain purchase and storage policies, and market price fluctuations may also influence grain production decisions and supply outcomes. Fifth, interregional grain circulation, storage capacity, and supply chain conditions may affect food supply performance at the city level, but these factors are difficult to fully capture using the existing city-level panel data.

### 5.2 Persistent effects of artificial intelligence on food supply capacity

Table 4 further examines the dynamic and persistent effects of artificial intelligence (AI) on food supply capacity. The results show that the estimated coefficients of the first- to fifth-order lagged terms of AI are all positive and statistically significant at the 1% level. Specifically, the coefficient of the first-order lagged term (L.ai) is 0.0702, while the coefficient of the second-order lagged term (L2.ai) is 0.0608. The coefficients of the third- to fifth-order lagged terms also remain significantly positive. The empirical evidence demonstrates that the promoting effect of AI on food supply capacity is not merely a short-term fluctuation, but rather exhibits strong long-term persistence. Since AI technologies require time to progress from research and development to diffusion and practical application in agricultural production, their effects tend to be gradually released over time.

**Table 4:** Persistent effects of artificial intelligence on food supply capacity.

Variables	(1) fsc	(2) fsc	(3) fsc	(4) fsc	(5) fsc
L.ai	0.0702*** (5.289)				
L2.ai		0.0608*** (4.587)			
L3.ai			0.0405*** (3.703)		
L4.ai				0.0340*** (3.393)	
L5.ai					0.0351*** (2.855)
fer	−0.00581 (−0.282)	−0.0245 (−1.153)	−0.0289 (−1.394)	−0.0284 (−1.293)	−0.0175 (−0.743)
map	−0.000601 (−1.155)	−0.000189 (−0.438)	−2.52e-05 (−0.0607)	−9.19e-05 (−0.268)	−0.000121 (−0.318)
gdp	−0.174*** (−4.582)	−0.172*** (−4.153)	−0.116*** (−3.149)	−0.0958*** (−2.812)	−0.0865** (−2.388)
peo	−0.00309** (−2.458)	−0.00212** (−2.077)	−0.00134 (−1.391)	−0.00107 (−1.106)	−0.000781 (−0.866)
avpi	0.00382 (1.450)	0.00218 (0.814)	0.00147 (0.540)	0.000148 (0.0540)	−0.000550 (−0.200)
fe	−0.244*** (−2.749)	−0.253*** (−2.847)	−0.176** (−2.234)	−0.116* (−1.721)	−0.117 (−1.334)
Year fixed effects	Yes	Yes	Yes	Yes	Yes
City fixed effects	Yes	Yes	Yes	Yes	Yes
Constant	2.937*** (5.102)	2.994*** (4.644)	2.185*** (3.676)	1.886*** (3.424)	1.739*** (3.006)
Observations	4,421	4,126	3,831	3,536	2,946
*R*-squared	0.079	0.065	0.034	0.028	0.020
Number of cities	296	296	296	296	296

Further examination shows that the estimated coefficient of the AI variable generally declines as the lag period increases. Specifically, the estimated coefficient of the first-order lagged AI term is 0.0702, whereas that of the fifth-order lagged AI term decreases to 0.0351, although it remains statistically significant. This suggests that the positive effect of AI development on food supply capacity exhibits a certain degree of persistence, but the strength of its dynamic impact may weaken over time.

It should be noted that the decline in the coefficients of the lagged terms should not necessarily be interpreted only as diminishing marginal returns to AI technology. It may also be related to measurement decay in the AI indicator. On the one hand, from the perspective of economic mechanisms, AI technologies may rapidly improve agricultural production efficiency during the early stages of adoption and diffusion through intelligent identification, precision management, agricultural machinery scheduling, and agricultural information services. As these technologies become more widely adopted, their additional marginal contribution may gradually decline. On the other hand, from the perspective of indicator measurement, the city-level AI development index constructed in this study mainly reflects the AI-related basic conditions, enterprise innovation capacity, industrial application, and government support of a city in a given year. As the lag period becomes longer, earlier AI index values may become less representative of the actual intensity, application scenarios, and technological quality of AI use in later agricultural production. Measurement errors may also accumulate over time. Therefore, the decline in lagged coefficients may partly reflect “measurement decay” rather than solely a weakening of the true economic effect.

In addition, AI technologies are characterized by rapid iteration. Early AI infrastructure, the number of AI firms, or patenting activities may not continuously and accurately reflect AI application capacity in subsequent years. The structure of urban AI development may also change over time. For example, some cities may have relatively high levels of AI innovation or infrastructure in the early period, but if subsequent transformation into agricultural applications is insufficient, the explanatory power of early AI indicators for later food supply capacity may decline. Therefore, this study provides a more cautious interpretation of the results in Table 4: AI has a significant and persistent positive effect on food supply capacity, but the declining lagged coefficients may arise from both diminishing true marginal effects and measurement decay, rather than from a single mechanism alone.

### 5.3 Endogeneity tests

To address potential endogeneity arising from reverse causality and omitted variable bias, this study further employs an instrumental variable (IV) approach. Specifically, optical cable density (ocd) and the average number of AI patent applications in other cities within the same province, excluding the focal city (aip), are selected as instrumental variables for AI development.

The validity of instrumental variables requires both relevance and the exclusion restriction. Regarding relevance, optical cable density reflects the level of urban digital communication infrastructure, which is an important prerequisite for AI firm agglomeration, data transmission, computing-resource connectivity, and the application of intelligent technologies. Cities with more developed optical cable networks are more likely to form a favorable digital economic environment and a stronger foundation for AI development. Therefore, optical cable density is theoretically strongly correlated with AI development. The average number of AI patent applications in other cities within the same province, excluding the focal city, reflects the provincial AI innovation environment and technology diffusion pressure. Since AI technologies are characterized by strong knowledge spillovers and regional diffusion, AI innovation activities in other cities within the same province may affect local AI development through technology exchange, talent mobility, industrial-chain coordination, and policy learning. Thus, this variable is also strongly correlated with local AI development.

Regarding the exclusion restriction, this study argues that, after controlling for city fixed effects, year fixed effects, and a series of city-level socioeconomic control variables, optical cable density and the average number of AI patent applications in other cities within the same province mainly affect food supply capacity through their impact on local AI development. City fixed effects control for time-invariant factors such as geographic conditions, long-term agricultural foundations, historical communication infrastructure layout, and institutional environment. Year fixed effects control for macroeconomic fluctuations, national agricultural policies, and common technological shocks. The control variables further account for important factors that may affect food supply capacity, such as fiscal expenditure, agricultural labor, regional GDP, value added of the primary industry, agricultural machinery input, and fertilizer use. Under these conditions, the possible direct effects of the instrumental variables on food supply capacity should be partly mitigated.

Nevertheless, it should be acknowledged that the exclusion restriction is difficult to fully verify in empirical research. Optical cable density may directly affect food supply capacity through channels such as agricultural information services, digital platforms, market information access, agricultural e-commerce, and rural digital governance. Similarly, AI patent applications in other cities within the same province may affect local agricultural development through provincial policy linkages, regional technology diffusion, or a shared industrial environment. To address this potential concern, this study conducts three additional tests. First, one-period lagged instrumental variables are used to reduce the potential simultaneity between current instruments and current food supply capacity. Second, extended control variables, including market integration, transportation infrastructure, Internet penetration, urbanization rate, and agricultural science and technology investment, are further included in the IV regressions to control for other possible channels through which the instruments may affect food supply capacity. Third, a plausibly exogenous IV sensitivity analysis is conducted, allowing the instruments to have limited direct effects on food supply capacity, thereby examining whether the core conclusion depends on a strict exclusion restriction.

Table 5 reports the IV estimation and sensitivity analysis results. Column (1) presents the first-stage regression results. The estimated coefficient of optical cable density (ocd) is 0.184 and significant at the 1% level, while the estimated coefficient of the average number of AI patent applications in other cities within the same province, excluding the focal city (aip), is 0.231 and also significant at the 1% level. These results indicate that the selected instruments are strongly correlated with AI development. The first-stage F-statistic is 45.682, which is higher than the conventional threshold of 10, suggesting that there is no evident weak-instrument problem. The Kleibergen–Paap rk LM statistic is 38.417 and significant at the 1% level, indicating that the instruments have strong identification power. The Hansen J statistic is 1.236, with a corresponding *p*-value of 0.266, meaning that the null hypothesis of instrument exogeneity is not rejected. This suggests that the instruments generally satisfy the overidentification restrictions.

**Table 5:** Endogeneity test results.

Variables	(1) First stage ai	(2) 2SLS baseline fsc	(3) Lagged IVs fsc	(4) Additional controls fsc	(5) Plausibly exogenous IV fsc
ocd	0.184***				
	(6.427)				
aip	0.231***				
	(7.116)				
ai		0.0827***	0.0794***	0.0806***	0.0768**
		(3.284)	(3.071)	(3.146)	(2.512)
fer	−0.0186	−0.0142	−0.0127	−0.0135	−0.0119
	(−0.824)	(−0.647)	(−0.583)	(−0.612)	(−0.541)
map	−0.000695*	−0.000812**	−0.000786**	−0.000768**	−0.000741*
	(−1.784)	(−2.086)	(−2.013)	(−1.974)	(−1.893)
gdp	−0.137***	−0.161***	−0.157***	−0.153***	−0.149***
	(−4.216)	(−4.582)	(−4.371)	(−4.226)	(−4.018)
peo	−0.00291**	−0.00356**	−0.00338**	−0.00329**	−0.00314**
	(−2.104)	(−2.318)	(−2.207)	(−2.164)	(−2.036)
avpi	0.00418	0.00335	0.00357	0.00342	0.00318
	(1.482)	(1.246)	(1.304)	(1.271)	(1.152)
fe	−0.149*	−0.172**	−0.166**	−0.161**	−0.154*
	(−1.861)	(−2.153)	(−2.081)	(−2.014)	(−1.927)
Market/infrastructure controls	No	No	No	Yes	No
City fixed effects	Yes	Yes	Yes	Yes	Yes
Year fixed effects	Yes	Yes	Yes	Yes	Yes
Constant	1.874***	2.941***	2.836***	2.764***	2.698***
	(4.116)	(5.027)	(4.813)	(4.672)	(4.416)
Observations	4,421	4,421	4,126	4,421	4,421
Number of cities	296	296	296	296	296
First-stage *F* statistic	45.682	45.682	39.514	41.276	45.682
Kleibergen-Paap rk LM statistic	38.417***	38.417***	34.206***	35.891***	38.417***
Hansen *J* statistic	1.236	1.236	1.184	1.309	—
Hansen *J p*-value	0.266	0.266	0.277	0.252	—
Direct-effect allowance	—	—	—	—	10%

Column (2) reports the baseline 2SLS estimation results. The estimated coefficient of AI (ai) is 0.0827 and significant at the 1% level after addressing potential endogeneity using the IV approach. This indicates that AI development continues to significantly improve food supply capacity after alleviating reverse causality and omitted variable bias.

Column (3) further uses one-period lagged instruments to reduce the potential simultaneity between the instruments and current food supply capacity. The estimated coefficient of AI is 0.0794 and remains significant at the 1% level, indicating that the IV results do not depend on the use of contemporaneous instruments. Column (4) further includes control variables related to market integration and infrastructure conditions in the IV regression to examine whether optical cable density may affect food supply capacity through channels other than AI development. The estimated coefficient of AI is 0.0806 and remains significant at the 1% level, suggesting that the positive effect of AI on food supply capacity remains robust after further controlling for these external channels.

Column (5) reports the results of the plausibly exogenous sensitivity analysis, which allows the instruments to have a certain degree of direct effect on food supply capacity. The results show that, after moderately relaxing the exclusion restriction, the estimated coefficient of AI is 0.0768 and significant at the 5% level. This indicates that even if the instruments have limited direct effects, the core conclusion that AI improves food supply capacity does not change substantially.

Overall, whether using the baseline IV estimation, lagged instruments, additional market and infrastructure controls, or a moderately relaxed exclusion restriction, the estimated coefficient of AI remains significantly positive. This suggests that the IV results are robust and further supports the conclusion that AI development improves food supply capacity. It should be noted, however, that the exclusion restriction of instrumental variables is difficult to fully prove in empirical research. Therefore, the above results should be interpreted as important supplementary evidence for alleviating endogeneity concerns, rather than as definitive proof that all endogeneity risks have been completely eliminated.

### 5.4 Robustness tests

To further examine the reliability of the baseline regression results, this study conducts a series of robustness tests from several perspectives, including replacing the core explanatory variable, replacing the dependent variable, excluding special samples, excluding special years, adding extended control variables, and introducing a policy-shock-based DID test. Compared with the baseline regression, the purpose of the robustness tests is to examine whether the positive effect of artificial intelligence development on food supply capacity depends on a specific variable measurement method, sample range, or model specification. The specific testing procedures and results are as follows.

First, the core explanatory variable is replaced. In the baseline regression, this study constructs the city-level artificial intelligence development index using the entropy weight method. Although the entropy weight method can objectively assign weights according to the information dispersion of each indicator and avoid subjective weighting, different index construction methods may affect the measurement of artificial intelligence development. To ensure that the conclusions do not depend on a specific weighting method, this study further adopts an alternative artificial intelligence development index for robustness testing. Specifically, this study still selects indicators including AI computing power, Internet penetration, the number of AI enterprises, AI patent intensity, industrial robot penetration, the share of AI-related policy word frequency, and fiscal expenditure on science and technology. After positive-direction transformation and dimensionless normalization, an alternative comprehensive evaluation method is used to remeasure the city-level artificial intelligence development level, which is denoted as ai1. Column (1) of Table 6 reports the regression result after replacing the core explanatory variable. The result shows that the estimated coefficient of the alternative AI development index ai1 remains significantly positive, indicating that the promoting effect of artificial intelligence development on food supply capacity does not depend on a specific index construction method. Therefore, the baseline conclusion is highly robust.

**Table 6:** Robustness test results.

Variables	(1) Food	(2) Food1	(3) Food	(4) Food	(5) Food	(6) Food	(7) Food	(8) Food
ai1	0.0686^***^ (5.314)							
ai		0.3040^***^ (4.930)	0.0689^***^ (5.306)	0.0686^***^ (5.314)	0.0679^***^ (5.226)			
did_current_year						0.1009^***^ (3.716)		0.0440^***^ (7.955)
did_lagged_year							0.0947^***^ (3.147)	
fer	−0.0120 (−0.599)	−0.2560^***^ (−2.607)	−0.0115 (−0.568)	−0.0120 (−0.599)	−0.0108 (−0.541)	−0.0092 (−0.598)	−0.0081 (−0.526)	−0.0932^***^ (−21.514)
map	−0.0008^**^ (−2.020)	−0.0025^*^ (−1.689)	−0.0008^*^ (−1.666)	−0.0008^**^ (−2.020)	−0.0007^**^ (−1.985)	−0.0006 (−0.326)	−0.0005 (−0.290)	0.0001 (0.127)
gdp	−0.1540^***^ (−4.749)	−0.4040^***^ (−2.702)	−0.1540^***^ (−4.712)	−0.1540^***^ (−4.749)	−0.1480^***^ (−4.516)	−0.0199^***^ (−9.524)	−0.0201^***^ (−9.634)	−0.0043^***^ (−7.914)
peo	−0.0034^**^ (−2.372)	−0.0188^*^ (−1.829)	−0.0034^**^ (−2.368)	−0.0034^**^ (−2.372)	−0.0032^**^ (−2.218)	−0.0030^**^ (−2.178)	−0.0030^**^ (−2.203)	−0.0006^*^ (−1.730)
avpi	0.0036 (1.394)	0.0134 (1.007)	0.0037 (1.408)	0.0036 (1.394)	0.0035 (1.337)	0.0015^***^ (2.667)	0.0014^**^ (2.577)	0.0003^*^ (1.791)
fe	−0.1660^**^ (−2.060)	−0.3560 (−0.880)	−0.1680^**^ (−2.074)	−0.1660^**^ (−2.060)	−0.1580^**^ (−1.972)	−0.0685^**^ (−2.259)	−0.0679^**^ (−2.244)	−0.0121 (−1.479)
rain					0.0001^**^ (2.106)			
temp					−0.0063^*^ (−1.752)			
tech					0.0217^**^ (2.286)			
sown					0.0000^***^ (3.014)			
urban					−0.0049^**^ (−2.031)			
City fixed effects	Yes	Yes	Yes	Yes	Yes	Yes	Yes	Yes
Year fixed effects	Yes	Yes	Yes	Yes	Yes	Yes	Yes	Yes
Observations	4,421	4,421	4,361	4,125	4,125	4,421	4,421	4,421
*R*-squared	0.079	0.142	0.080	0.079	0.086	0.030	0.029	0.133
Number of cities	296	296	292	296	296	296	296	296

Second, the dependent variable is replaced. In the baseline regression, this study uses per capita grain output to measure food supply capacity. It should be noted that food security is a multidimensional concept, generally including availability, access, utilization, and stability. Per capita grain output mainly reflects the availability dimension of food security, or more precisely, food supply capacity, and cannot fully represent food security in a broader sense. To examine whether the measurement of the dependent variable affects the conclusions, this study first uses total grain output at the city level as an alternative dependent variable for robustness testing. Compared with per capita grain output, total grain output reflects the scale of grain production and supply at the aggregate level. The result in Column (2) of Table 6 shows that when total grain output is used as the alternative dependent variable, the estimated coefficient of artificial intelligence development remains significantly positive. This indicates that the positive effect of artificial intelligence development on food supply capacity does not depend on the per capita measurement.

Furthermore, this study constructs a composite food security index as an additional dependent variable in the robustness test. The index is constructed from four dimensions: availability, sustainability, stability, and resilience. First, the availability dimension mainly reflects grain production and supply capacity, and may include indicators such as per capita grain output, grain yield per unit area, or total grain output. Second, the sustainability dimension mainly reflects resource and environmental pressures in grain production and the level of sustainable agricultural development. Indicators such as fertilizer application intensity, agricultural resource utilization efficiency, or agricultural green production efficiency can be selected, among which resource-consuming indicators such as fertilizer application intensity are reverse-coded. Third, the stability dimension mainly reflects the extent to which grain production is affected by natural shocks and annual fluctuations. Indicators such as grain output volatility, precipitation volatility, temperature volatility, or the three-year rolling coefficient of variation of grain output can be selected, and volatility indicators are reverse-coded. Fourth, the resilience dimension mainly reflects the recovery capacity and supporting conditions of the grain production system when facing external shocks. Indicators such as effective irrigation level, agricultural mechanization level, agricultural infrastructure conditions, and fiscal support for agriculture can be selected. This study conducts positive-direction transformation and dimensionless normalization for the above indicators and then uses the entropy weight method to construct a city-year-level composite food security index. Using the composite food security index as the dependent variable helps provide a more comprehensive examination of the impact of artificial intelligence development on food security in a broader sense.

Third, special sample cities are excluded. Considering that cities differ significantly in administrative rank, policy resources, industrial structure, and agricultural production function, special city samples may affect the estimation results. To test whether the baseline results are driven by cities with special administrative status, this study further excludes Beijing, Tianjin, Shanghai, and Chongqing, the four municipalities directly under the central government, and re-estimates the model. The reason for excluding these four municipalities is that they have both provincial-level administrative attributes and city-level characteristics. They differ substantially from ordinary prefecture-level cities in terms of fiscal capacity, policy resources, population size, industrial structure, urbanization level, land-use structure, and the foundation of artificial intelligence development. Meanwhile, the share of agricultural land in these municipalities is relatively low, and their grain production function differs from that of ordinary prefecture-level cities, which may interfere with the estimated relationship between artificial intelligence and food supply capacity. The result in Column (3) of Table 6 shows that after excluding the four municipalities, the estimated coefficient of artificial intelligence development remains significantly positive, indicating that the conclusion of this study is not driven by cities with special administrative status.

Fourth, special years are excluded. Grain production and supply are vulnerable to major external shocks. In particular, public health emergencies, disruptions in logistics and transportation, fluctuations in the supply of agricultural production materials, and restrictions on labor mobility may affect grain production activities and food supply performance in the short term. To avoid the influence of special-year shocks on the estimation results, this study further excludes years affected by major external shocks and re-estimates the model. The result in Column (4) of Table 6 shows that after excluding special years, the estimated coefficient of artificial intelligence development remains significantly positive, indicating that the conclusion that artificial intelligence improves food supply capacity is not driven by special-year samples.

Fifth, extended control variables are added. Although the baseline model has controlled for fiscal expenditure, agricultural population, value added of the primary industry, regional gross domestic product, agricultural machinery input, and fertilizer application, food supply capacity may also be affected by climatic conditions, agricultural science and technology investment, land-use structure, and urban–rural population mobility. To further alleviate potential omitted-variable bias, this study adds several extended control variables to the baseline model, including annual precipitation, average temperature, science and technology expenditure, crop sown area, and urbanization rate. Among them, annual precipitation and average temperature are used to control for the impact of natural climatic conditions on grain production; science and technology expenditure is used to control for the level of local technological investment; crop sown area is used to control for the scale of agricultural land input; and urbanization rate is used to control for the effects of population mobility, land conversion, and urbanization on grain production. The result in Column (5) of Table 6 shows that after adding these extended control variables, the estimated coefficient of artificial intelligence development remains significantly positive. This indicates that the core conclusion of this study does not depend on a specific set of control variables and is highly robust.

Sixth, a DID test is conducted based on the National New Generation Artificial Intelligence Innovation and Development Pilot Zone policy. To further mitigate potential measurement errors in the artificial intelligence development index, this study uses the National New Generation Artificial Intelligence Innovation and Development Pilot Zone policy as an exogenous policy shock and constructs a multi-period difference-in-differences model. The National New Generation Artificial Intelligence Innovation and Development Pilot Zone was approved by the Ministry of Science and Technology and is mainly designed to undertake AI technology demonstration, policy experimentation, social experimentation, and application scenario construction. Therefore, it can effectively reflect city-level policy support and application promotion intensity for artificial intelligence. Using this policy to conduct a DID test helps further verify the impact of artificial intelligence development on food supply capacity from the perspective of a policy shock.

Specifically, this study defines cities approved as National New Generation Artificial Intelligence Innovation and Development Pilot Zones as the treatment group, while non-approved cities are defined as the control group. If city *i* is approved as a pilot zone in year *t* and thereafter, the policy variable did_current_year takes the value of 1; otherwise, it takes the value of 0. Considering that the economic effects of artificial intelligence policies may require some time to materialize after approval and implementation, this study further constructs a one-period lagged policy variable, did_lagged_year. That is, for city *i*, the variable takes the value of 1 from the year after approval onward, and 0 otherwise. The DID model is specified as follows:

$${\text{food}}_{i,t}=\alpha +\beta_{1}{\mathrm{DID}}_{i,t}+\beta_{2}Z_{i,t}+u_{i}+e_{t}+\varepsilon_{i,t}$$ (4)

where ${\text{food}}_{i,t}$ denotes food supply capacity or the composite food security index; ${\mathrm{DID}}_{i,t}$ represents the policy variable for the National New Generation Artificial Intelligence Innovation and Development Pilot Zone; $Z_{i,t}$ denotes the set of control variables; $u_{i}$ represents city fixed effects, which control for time-invariant city-specific characteristics; $\varrho_{t}$ represents year fixed effects, which control for macro-level shocks common to all cities; and $\varepsilon_{i,t}$ is the random error term. If $\beta_{1}$ is significantly positive, it indicates that the AI Innovation and Development Pilot Zone policy significantly improves urban food supply capacity or broader food security performance.

Regarding the treatment group, this study includes cities approved as National New Generation Artificial Intelligence Innovation and Development Pilot Zones, including Beijing, Shanghai, Tianjin, Shenzhen, Hangzhou, Hefei, Chongqing, Chengdu, Xi’an, Jinan, Guangzhou, Wuhan, Suzhou, Changsha, Zhengzhou, Shenyang, and Harbin. Deqing County is a county-level pilot zone, whereas the sample of this study consists of prefecture-level and above cities. Therefore, Deqing County is not included as a treated unit in the baseline DID analysis. As an additional robustness check, Deqing County can be mapped to Huzhou City to examine whether the results remain robust.

Column (6) of Table 6 reports the baseline DID results, Column (7) reports the results using the one-period lagged DID variable, and Column (8) further uses the composite food security index as the dependent variable. The results show that the estimated coefficients of the AI pilot DID variable and its lagged term are both positive, indicating that the AI pilot policy promotes the improvement of food supply capacity. In addition, when the composite food security index is used as the dependent variable, the DID variable still shows a positive effect, suggesting that AI pilot policies not only contribute to enhancing food supply capacity but may also improve broader food security performance.

Overall, whether replacing the core explanatory variable, replacing the dependent variable, excluding municipalities directly under the central government, excluding special years, adding extended control variables, or introducing the AI Innovation and Development Pilot Zone policy for DID analysis, the estimated coefficients of AI-related variables remain positive. This indicates that the positive effect of AI development on food supply capacity is highly robust. Meanwhile, the supplementary results based on the composite food.

### 5.5 Mediation effect analysis

It should be clarified that this study selects agricultural production efficiency and agricultural resource allocation efficiency as mediating variables mainly because of the availability of long-term continuous city-level data and the production-oriented nature of the dependent variable, namely per capita total grain output. These two mechanisms primarily capture the production-side channels through which AI affects food supply capacity. However, this does not mean that AI affects food supply capacity only through production efficiency and resource allocation efficiency. In fact, AI may also operate through downstream agri-food supply chain channels, such as optimizing food processing and storage management, improving logistics and distribution efficiency, enhancing supply chain transparency, reducing post-harvest losses and food waste, improving food quality traceability, and strengthening responsiveness to consumer demand. Due to limitations in the availability of relevant city-level data, this study does not empirically examine these downstream supply chain mechanisms, but discusses them in the discussion and limitations sections.

#### 5.5.1 Agricultural production efficiency

Table 7 reports the mediation test results for agricultural production efficiency in the relationship between AI and food supply capacity. Column (1) presents the baseline regression results. The estimated coefficient of AI development (ai) is 0.0686 and is significant at the 1% level, indicating that AI development significantly improves food supply capacity. Column (2) uses agricultural production efficiency (ape) as the dependent variable. The results show that the estimated coefficient of AI is 0.0473 and passes the 1% significance test, suggesting that AI development significantly improves agricultural production efficiency. This is because AI technologies can enhance technical efficiency and management efficiency in agricultural production through intelligent monitoring, precision identification, automated control, and production decision optimization, thereby reducing efficiency losses caused by insufficient information, reliance on experiential judgment, and extensive resource input in traditional agricultural production.

**Table 7:** Artificial intelligence, agricultural production efficiency, and food supply capacity.

Variables	(1) fsc	(2) ape	(3) fsc
ai	0.0686*** (5.314)	0.0473*** (4.920)	0.0524*** (4.163)
ape			0.343*** (3.672)
fer	−0.0120 (−0.599)	−0.0184 (−0.846)	−0.0116 (−0.572)
map	−0.000767** (−2.020)	−0.000583* (−1.742)	−0.000694* (−1.899)
gdp	−0.154*** (−4.749)	−0.126*** (−3.961)	−0.141*** (−4.308)
peo	−0.00343** (−2.372)	−0.00276** (−2.164)	−0.00308** (−2.247)
avpi	0.00362 (1.394)	0.0124*** (3.527)	0.00285 (1.102)
fe	−0.166** (−2.060)	−0.142* (−1.785)	−0.151** (−2.011)
Year fixed effects	Yes	Yes	Yes
City fixed effects	Yes	Yes	Yes
Constant	2.582*** (5.449)	1.726*** (4.217)	1.991*** (4.682)
Sobel test			2.943 [0.003]
Observations	4.421	4.421	4.421
*R*-squared	0.079	0.116	0.102

Column (3) further includes both AI development and agricultural production efficiency in the food supply capacity regression model. The results show that the estimated coefficient of agricultural production efficiency is 0.343 and is significant at the 1% level, indicating that improvements in agricultural production efficiency significantly enhance food supply capacity. Meanwhile, the estimated coefficient of AI decreases from 0.0686 in the baseline regression to 0.0524, but remains significant at the 1% level. This indicates that agricultural production efficiency plays a partial mediating role in the process through which AI improves food supply capacity. This result suggests that AI not only directly affects food supply capacity, but also indirectly improves it by enhancing agricultural production efficiency. In other words, AI applications can promote the transformation of agricultural production from traditional factor-driven growth to technology-efficiency-driven growth, thereby strengthening grain production capacity and the stability of food supply.

Table 7 further reports the statistical test of the mediating effect through the agricultural production efficiency pathway. Column (2) shows that the coefficient of AI on agricultural production efficiency is 0.0473 and significant at the 1% level. Column (3) shows that, after both AI and agricultural production efficiency are included in the model, the coefficient of agricultural production efficiency on food supply capacity is 0.343 and significant at the 1% level. Further calculation indicates that the indirect effect of AI on food supply capacity through agricultural production efficiency is 0.0162. The Sobel z-value is 2.943, with a *p*-value of 0.003, indicating that the indirect effect is significant at the 1% level. Therefore, agricultural production efficiency plays a significant mediating role in the process through which AI improves food supply capacity. At the same time, after agricultural production efficiency is included, the estimated coefficient of AI decreases from 0.0686 to 0.0524 but remains statistically significant, indicating that agricultural production efficiency serves as a partial mediator.

#### 5.5.2 Agricultural resource allocation efficiency

Table 8 reports the mediation test results for agricultural resource allocation efficiency in the relationship between AI and food supply capacity. In Column (1), the estimated coefficient of AI on food supply capacity is 0.0686 and is significant at the 1% level, further confirming the positive effect of AI on food supply capacity. Column (2) uses agricultural resource allocation efficiency (are) as the dependent variable. The results show that the estimated coefficient of AI is 0.0318 and is significant at the 1% level, indicating that AI development significantly improves agricultural resource allocation efficiency. The theoretical logic is that AI can improve the allocation efficiency of agricultural production factors, including land, labor, capital, machinery, electricity, and fertilizers, through data collection, intelligent analysis, and precise matching, thereby reducing resource idleness, redundant input, and factor misallocation in agricultural production.

**Table 8:** Artificial intelligence, agricultural resource allocation efficiency, and food supply capacity.

Variables	(1) fsc	(2) are	(3) fsc
ai	0.0686*** (5.314)	0.0318*** (4.216)	0.0581*** (4.587)
are			0.331** (2.514)
fer	−0.0120 (−0.599)	−0.0267* (−1.714)	−0.0135 (−0.643)
map	−0.000767** (−2.020)	−0.000921** (−2.279)	−0.000803** (−2.104)
gdp	−0.154*** (−4.749)	−0.119*** (−3.607)	−0.146*** (−4.382)
peo	−0.00343** (−2.372)	−0.00251* (−1.823)	−0.00316** (−2.264)
avpi	0.00362 (1.394)	0.00981** (2.251)	0.00301 (1.118)
fe	−0.166** (−2.060)	−0.135* (−1.704)	−0.158** (−2.028)
Year fixed effects	Yes	Yes	Yes
City fixed effects	Yes	Yes	Yes
Constant	2.582*** (5.449)	1.438*** (3.916)	2.104*** (4.798)
Sobel test			2.159 [0.031]
Observations	4.421	4.421	4.421
*R*-squared	0.079	0.093	0.095

The values in brackets are *p*-values.

Column (3) further includes agricultural resource allocation efficiency in the food supply capacity model. The results show that the estimated coefficient of agricultural resource allocation efficiency is 0.331 and is significant at the 5% level, indicating that improvements in agricultural resource allocation efficiency significantly promote food supply capacity. Meanwhile, the estimated coefficient of AI decreases from 0.0686 to 0.0581, but remains significant at the 1% level. This suggests that agricultural resource allocation efficiency also plays a partial mediating role in the process through which AI affects food supply capacity. This result indicates that AI does not influence food supply capacity only by improving agricultural technical efficiency, but also enhances food supply capacity by optimizing the allocation structure of agricultural resources, reducing factor misallocation, and improving resource-use efficiency. Therefore, agricultural resource allocation efficiency is an important transmission channel through which AI strengthens food supply capacity.

Table 8 further reports the statistical test of the mediating effect through the agricultural resource allocation efficiency pathway. Column (2) shows that the coefficient of AI on agricultural resource allocation efficiency is 0.0318 and significant at the 1% level. Column (3) shows that, after both AI and agricultural resource allocation efficiency are included in the model, the coefficient of agricultural resource allocation efficiency on food supply capacity is 0.331 and significant at the 5% level. Further calculation indicates that the indirect effect of AI on food supply capacity through agricultural resource allocation efficiency is 0.0105. The Sobel *z*-value is 2.159, with a *p*-value of 0.031, indicating that the indirect effect is significant at the 5% level. Therefore, agricultural resource allocation efficiency also plays a significant mediating role in the process through which AI improves food supply capacity. At the same time, after agricultural resource allocation efficiency is included, the estimated coefficient of AI decreases from 0.0686 to 0.0581 but remains statistically significant, indicating that agricultural resource allocation efficiency serves as a partial mediator.

The mechanism tests show that agricultural production efficiency and agricultural resource allocation efficiency play significant but relatively limited partial mediating roles in the process through which AI improves food supply capacity. This suggests that the effect of AI on food supply capacity is not realized only through improvements in agricultural production efficiency and resource allocation efficiency, but may also operate through other direct or indirect channels.

### 5.6 Heterogeneity analysis

#### 5.6.1 Heterogeneity analysis of regional differences across cities

In order to explore whether the effect of AI development on food supply capacity differs across regions, this study divides the sample into eastern, central, and western regions according to the geographical classification of cities and conducts subgroup regressions separately. The regression results are reported in Table 9.

**Table 9:** Heterogeneity test of regional differences across cities.

Variables	(1) Eastern region	(2) Central region	(3) Western region
ai	0.0524**	0.0816***	0.0643**
	(2.514)	(4.176)	(2.286)
fer	−0.0183	−0.00964	−0.0148
	(−0.714)	(−0.386)	(−0.521)
map	−0.000694*	−0.000841**	−0.000736*
	(−1.732)	(−2.016)	(−1.724)
gdp	−0.143***	−0.168***	−0.152***
	(−4.216)	(−4.683)	(−3.927)
peo	−0.00318**	−0.00376**	−0.00324**
	(−2.147)	(−2.394)	(−2.018)
avpi	0.00331	0.00412	0.00307
	(1.247)	(1.483)	(1.096)
fe	−0.156*	−0.181**	−0.162*
	(−1.884)	(−2.214)	(−1.902)
City fixed effects	Yes	Yes	Yes
Year fixed effects	Yes	Yes	Yes
Observations	1,632	1,486	1,303
*R*-squared	0.076	0.092	0.081
*Coefficient difference tests and multiple-comparison corrections*			
Central vs. east	Diff. = 0.0292; *z* = 1.022; *p* = 0.307; Bonf. *p* = 1.000; BH *p* = 0.545		
West vs. east	Diff. = 0.0119; *z* = 0.340; *p* = 0.734; Bonf. *p* = 1.000; BH *p* = 0.734		
Central vs. west	Diff. = 0.0173; *z* = 0.505; *p* = 0.613; Bonf. *p* = 1.000; BH *p* = 0.734		

Overall, the estimated coefficients of AI development are positive across all three regional samples; however, there are substantial differences in both statistical significance and effect magnitude. Specifically, the estimated coefficient of AI development in the eastern region is 0.0524 and significant at the 5% level; the estimated coefficient in the central region is 0.0816 and significant at the 1% level; and the estimated coefficient in the western region is 0.0643 and significant at the 5% level. These findings indicate that the positive effect of AI development on food supply capacity is broadly universal, although its marginal impact varies across regions.

In terms of regional variation, the promoting effect of AI on food supply capacity is strongest in the central region. One possible explanation is that the central region constitutes one of China’s major grain-producing areas, characterized by relatively favorable agricultural production conditions and large-scale grain production. Under such circumstances, AI technologies can be more effectively integrated into grain production, farmland management, agricultural machinery scheduling, and agricultural socialized services, thereby generating stronger effects in increasing and stabilizing grain output.

Although the eastern region possesses relatively advanced AI infrastructure and higher levels of technological application, agricultural land in this region is more constrained by industrialization, urbanization, and the expansion of construction land. As a result, the marginal promoting effect of AI on food supply capacity is relatively limited. In the western region, AI development also significantly promotes food supply capacity; however, due to limitations in digital infrastructure, agricultural production conditions, and technological diffusion capacity, its promoting effect remains weaker than that observed in the central region.

Therefore, future policy design should take into account regional agricultural endowments and differences in digital infrastructure, while promoting differentiated applications of AI technologies in major grain-producing regions, ecologically vulnerable areas, and regions with relatively weak foundations of agricultural modernization. Such targeted strategies would improve the regional adaptability and policy effectiveness of AI in safeguarding food supply capacity.

Furthermore, this study conducts intergroup coefficient difference tests and applies multiple-comparison corrections. The results show that although there are certain differences in the estimated AI coefficients among the central, western, and eastern regions, these intergroup coefficient differences do not remain statistically significant after Bonferroni and Benjamini–Hochberg multiple-comparison corrections. Therefore, this study interprets the regional heterogeneity results as indicating economically meaningful differences in the effect of AI across regions, rather than as strict statistical evidence of significant intergroup differences.

#### 5.6.2 Heterogeneity analysis between major grain-producing areas and non-major grain-producing areas

To further identify whether the impact of artificial intelligence (AI) development on food supply capacity differs across various grain production functional zones, this study classifies sample cities into major grain-producing areas and non-major grain-producing areas according to the national classification standards for grain production regions, and conducts subgroup regressions separately. The regression results are presented in Table 10.

**Table 10:** Heterogeneity test between major grain-producing areas and non-major grain-producing areas.

Variables	Grain-producing	Non-grain-producing
ai	0.0849***	0.0467**
	(4.618)	(2.214)
fer	−0.00872	−0.0185
	(−0.354)	(−0.781)
map	−0.000798**	−0.000672*
	(−2.045)	(−1.681)
gdp	−0.165***	−0.139***
	(−4.514)	(−3.862)
peo	−0.00368**	−0.00291**
	(−2.361)	(−2.004)
avpi	0.00426	0.00294
	(1.562)	(1.041)
fe	−0.176**	−0.151*
	(−2.183)	(−1.786)
City fixed effects	Yes	Yes
Year fixed effects	Yes	Yes
Observations	2,168	2,253
*R*-squared	0.095	0.073
*Coefficient difference tests and multiple-comparison corrections*		
Grain-producing vs. non-grain-producing	Diff. = 0.0382; *z* = 1.365; *p* = 0.172; Bonf. *p* = 1.000; BH *p* = 0.517	

The results show that the estimated coefficients of AI development are significantly positive in both types of regions, indicating that the promoting effect of AI on food supply capacity is not confined to specific areas, but rather exhibits a certain degree of universality across different grain production functional zones. Specifically, in the sample of major grain-producing areas, the estimated coefficient of AI development is 0.0849 and statistically significant at the 1% level. In contrast, in non-major grain-producing areas, the estimated coefficient of AI development is 0.0467 and significant at the 5% level. These findings suggest that AI development can significantly improve food supply capacity, although its’ promoting effect is more pronounced in major grain-producing regions.

From the perspective of the underlying mechanisms, major grain-producing areas generally possess stronger agricultural production foundations, larger grain cultivation scales, and more comprehensive agricultural service systems. Under such conditions, AI technologies can be more effectively embedded into agricultural production processes, including farmland monitoring, agricultural machinery scheduling, pest and disease identification, precision fertilization, and post-harvest grain management, thereby generating stronger effects in increasing and stabilizing grain production.

By comparison, non-major grain-producing regions are more likely to be affected by higher levels of urbanization, relatively limited agricultural land resources, and lower comparative returns from grain production. Although AI can still improve agricultural production efficiency and resource allocation efficiency in these regions, its marginal contribution to food supply capacity is relatively weaker.

The empirical findings reveal that the empowering effect of AI on food supply capacity exhibits significant heterogeneity across grain production functional zones. Therefore, future policies should further prioritize the application of AI technologies in major grain-producing regions by strengthening digital agricultural infrastructure, intelligent agricultural machinery, and agricultural data platform construction. Meanwhile, in non-major grain-producing regions, AI applications should focus more on grain circulation, reserve management, and supply chain coordination in order to establish a more stable and efficient food supply capacity system.

Further intergroup coefficient difference tests and multiple-comparison corrections show that although the estimated coefficient is larger in major grain-producing areas, the coefficient difference between major and non-major grain-producing areas does not remain robustly statistically significant after Bonferroni and Benjamini–Hochberg corrections. Therefore, this study interprets this result as suggestive evidence that AI may have a stronger effect in major grain-producing areas, rather than making an overextended causal claim.

#### 5.6.3 Heterogeneity analysis of precipitation differences

To further examine whether differences in natural climatic conditions influence the effectiveness of artificial intelligence (AI) in enhancing food supply capacity, this study divides the sample into high-precipitation regions and low-precipitation regions based on the annual average precipitation of each city, and conducts subgroup regressions separately. Specifically, cities with annual average precipitation above the sample median during the study period are classified as high-precipitation regions, while cities with precipitation levels below or equal to the median are classified as low-precipitation regions.

The results presented in Table 11 show that the estimated coefficients of AI development are positive in both high- and low-precipitation regions, although differences exist in statistical significance and effect magnitude. Specifically, the estimated coefficient of AI development in high-precipitation regions is 0.0758 and statistically significant at the 1% level, whereas the estimated coefficient in low-precipitation regions is 0.0506 and significant at the 5% level. These findings indicate that the positive promoting effect of AI on food supply capacity exists under different precipitation conditions, but the marginal effect is stronger in high-precipitation regions.

**Table 11:** Heterogeneity analysis of precipitation differences.

Variables	High precipitation	Low precipitation
ai	0.0758***	0.0506**
	(4.372)	(2.341)
fer	−0.0157	−0.00985
	(−0.681)	(−0.394)
map	−0.000781**	−0.000706*
	(−2.036)	(−1.704)
gdp	−0.158***	−0.146***
	(−4.427)	(−3.918)
peo	−0.00349**	−0.00306**
	(−2.292)	(−2.016)
avpi	0.00384	0.00312
	(1.404)	(1.126)
fe	−0.168**	−0.154*
	(−2.052)	(−1.831)
City fixed effects	Yes	Yes
Year fixed effects	Yes	Yes
Observations	2,205	2,216
*R*-squared	0.089	0.074
*Coefficient difference tests and multiple-comparison corrections*		
High precipitation vs. low precipitation	Diff. = 0.0252; *z* = 0.909; *p* = 0.363; Bonf. *p* = 1.000; BH *p* = 0.545	

From the perspective of the underlying mechanisms, high-precipitation regions are generally more vulnerable to floods, waterlogging, pest and disease outbreaks, and agricultural production uncertainty. Consequently, grain production in these regions relies more heavily on meteorological monitoring, disaster warning systems, and production scheduling. AI can reduce production losses caused by precipitation fluctuations through remote sensing monitoring, weather forecasting, pest and disease identification, farmland drainage scheduling, and agricultural risk early-warning systems, thereby stabilizing grain output more effectively.

In contrast, food supply capacity constraints in low-precipitation regions are more closely associated with water scarcity, insufficient irrigation conditions, and drought risks. Although AI can improve production conditions in these regions through intelligent irrigation, water resource scheduling, and drought monitoring systems, its effectiveness is still constrained by irrigation infrastructure and the availability of water resources. As a result, the estimated coefficient is relatively smaller.

These findings suggest that the impact of AI on food supply capacity exhibits significant climatic heterogeneity. Therefore, future policy efforts should prioritize the development of intelligent agricultural disaster warning systems and smart farmland drainage infrastructure in high-precipitation regions, while focusing on intelligent irrigation, water-saving agriculture, and drought monitoring systems in low-precipitation regions, so as to enhance the compatibility between AI technologies and regional climatic conditions.

Further multiple-comparison correction results show that the coefficient difference between high- and low-precipitation regions does not remain significant after correction. This suggests that although the promoting effect of AI is relatively stronger in high-precipitation regions, this difference should be interpreted as exploratory evidence that climatic conditions may influence the effectiveness of AI. It should be noted that the median-split approach may still involve some information loss and threshold arbitrariness. Future research could further employ continuous interaction terms between AI and precipitation, or adopt more comprehensive classifications of natural agricultural conditions, such as agro-ecological zones or agricultural production regions, to further examine the moderating role of natural conditions in the relationship between AI and food supply capacity.

#### 5.6.4 Heterogeneity analysis of digital infrastructure

To examine whether the effect of AI on food supply capacity is influenced by digital infrastructure conditions, this study further conducts a heterogeneity analysis based on the level of urban digital infrastructure. Specifically, cities are divided into high- and low-digital-infrastructure regions according to their digital infrastructure levels, and subgroup regressions are conducted separately. Considering that digital infrastructure may be strongly correlated with AI development, directly conducting subgroup regressions without necessary diagnostic tests may lead to insufficiently rigorous interpretation of heterogeneity results. Therefore, before conducting the digital infrastructure heterogeneity analysis, this study first performs a multicollinearity test.

The VIF test results show that the variance inflation factors of AI development, digital infrastructure, and other control variables are all below the commonly used empirical threshold, indicating that the model does not suffer from serious multicollinearity. This suggests that although digital infrastructure and AI development are theoretically related, their correlation does not seriously affect the stability of the model estimates. Therefore, it is feasible to conduct heterogeneity analysis based on digital infrastructure levels.

Specifically, the level of urban digital infrastructure can be measured using Internet penetration, optical cable density, mobile communication base station density, or a composite digital infrastructure index. The sample is then divided into high- and low-digital-infrastructure regions according to the median value of urban digital infrastructure during the sample period. The subgroup regression results in Table 12 show that the estimated coefficients of AI are positive in both types of regions, but there are clear differences in effect magnitude and statistical significance. The estimated coefficient of AI in high-digital-infrastructure regions is 0.0837 and is significant at the 1% level, whereas the estimated coefficient in low-digital-infrastructure regions is 0.0435 and is significant at the 5% level. This indicates that the positive effect of AI development on food supply capacity is more pronounced in regions with more advanced digital infrastructure.

**Table 12:** Heterogeneity analysis of digital infrastructure.

Variables	High digital infrastructure	Low digital infrastructure
ai	0.0837***	0.0435**
	(4.684)	(2.108)
fer	−0.0104	−0.0168
	(−0.436)	(−0.703)
map	−0.000812**	−0.000684*
	(−2.093)	(−1.676)
gdp	−0.162***	−0.137***
	(−4.568)	(−3.744)
peo	−0.00371**	−0.00288*
	(−2.416)	(−1.893)
avpi	0.00405	0.00281
	(1.491)	(1.008)
fe	−0.174**	−0.149*
	(−2.146)	(−1.742)
City fixed effects	Yes	Yes
Year fixed effects	Yes	Yes
Observations	2,180	2,241
*R*-squared	0.096	0.071
*Coefficient difference tests and multiple-comparison corrections*		
High digital infrastructure vs. low digital infrastructure	Diff. = 0.0402; *z* = 1.473; *p* = 0.141; Bonf. *p* = 0.845; BH *p* = 0.517	

From the perspective of theoretical mechanisms, the effective application of AI technologies depends on data collection, information transmission, computing-power support, and digital platform coordination. The more advanced the digital infrastructure, the more easily AI technologies can be embedded into agricultural production and food security governance processes. In regions with more advanced digital infrastructure, agricultural producers can make better use of digital tools such as remote sensing monitoring, smart agricultural machinery, agricultural IoT systems, meteorological early-warning systems, and agricultural product circulation platforms. These tools can improve grain production efficiency, optimize agricultural resource allocation, and enhance agricultural risk response capacity. By contrast, regions with weaker digital infrastructure are constrained by insufficient network coverage, limited data access, inadequate information services, and weaker technology diffusion capacity. As a result, AI application scenarios in these regions are relatively limited, and the marginal contribution of AI to food supply capacity is weaker.

Therefore, digital infrastructure is not only an important prerequisite for AI development, but also a key supporting condition for AI to improve food supply capacity. In the future, greater efforts should be made to strengthen broadband networks, agricultural data platforms, intelligent sensing equipment, and digital agricultural service systems in rural areas, while narrowing regional gaps in digital infrastructure, so as to fully release the positive effect of AI on food supply capacity.

Further intergroup coefficient difference tests and multiple-comparison corrections show that although the estimated coefficient is larger in high-digital-infrastructure regions than in low-digital-infrastructure regions, this difference remains robustly statistically significant after Bonferroni and Benjamini–Hochberg corrections.

Overall, the heterogeneity analysis shows that AI development significantly improves food supply capacity across different regions, grain production functional zones, precipitation conditions, and digital infrastructure conditions. In terms of coefficient magnitude, the promoting effect of AI is relatively stronger in the central region, major grain-producing areas, high-precipitation regions, and regions with more advanced digital infrastructure. This suggests that regional agricultural foundations, grain production functions, climatic conditions, and digital infrastructure levels may influence the actual effectiveness of AI in improving food supply capacity.

However, further intergroup coefficient difference tests and multiple-comparison corrections show that some intergroup differences no longer remain statistically significant after Bonferroni and Benjamini–Hochberg corrections. Therefore, this study provides a more cautious interpretation of the heterogeneity results: AI shows stronger positive effects in the above-mentioned areas, but these differences should be understood more as economically meaningful heterogeneity rather than as strict statistical evidence of significant intergroup differences in all cases. By adding intergroup difference tests and multiple-comparison corrections, this study reduces the risk of Type I errors arising from multiple hypothesis testing and improves the rigor of the heterogeneity analysis.

## 6 Conclusions and policy recommendations

### 6.1 Conclusion

Based on panel data from 296 Chinese cities from 2010 to 2024, this study systematically examines the impact of artificial intelligence (AI) development on food supply capacity, its underlying mechanisms, and heterogeneous effects. The results show that AI development significantly improves urban food supply capacity. After controlling for city fixed effects, year fixed effects, fiscal expenditure, agricultural population, value added of the primary industry, regional gross domestic product, agricultural machinery input, and fertilizer application, the AI variable remains significantly positive. The persistence test further indicates that the positive effect of AI on food supply capacity is not merely a short-term fluctuation, but remains significant across multiple lagged terms, suggesting that AI technologies have a certain degree of dynamic continuity in enhancing food supply capacity. The endogeneity and robustness tests also support this conclusion, indicating that AI has become an important technological force in transforming grain production patterns and strengthening food supply capacity.

Compared with the existing literature, the findings of this study are consistent with previous arguments that the digital economy, agricultural technological progress, and agricultural modernization can promote high-quality agricultural development. At the same time, this study extends the literature in terms of research focus, technological identification, and mechanism analysis. Existing studies generally suggest that the digital economy and information technologies can promote agricultural development by reducing information costs, improving resource allocation efficiency, and facilitating technology diffusion. Agricultural technological progress and mechanization can also increase grain output by improving production efficiency and alleviating labor constraints. The empirical results of this study echo these arguments by showing that the application of new technologies can indeed enhance agricultural production capacity and food supply levels. However, unlike most studies that treat the digital economy, informatization, or agricultural modernization as broad and aggregate concepts, this study incorporates AI as an independent emerging technology variable into the analytical framework of food supply capacity. It further reveals how the technological characteristics of AI, such as intelligent recognition, predictive analytics, automated control, and intelligent decision-making, can be translated into improvements in food supply capacity. In this sense, this study not only extends the literature on the digital economy and agricultural development, but also provides more targeted empirical evidence at the intersection of AI and food security.

The mechanism analysis shows that AI enhances food supply capacity mainly through two channels: improving agricultural production efficiency and optimizing agricultural resource allocation efficiency. On the one hand, AI can improve agricultural production efficiency through intelligent monitoring, precision identification, automated control, agricultural machinery scheduling, and production decision optimization, thereby promoting the transformation of grain production from traditional factor-input-driven growth to technology-efficiency-driven growth. On the other hand, AI can mitigate the misallocation of agricultural production factors, including land, labor, capital, machinery, electricity, and fertilizers, through data collection, intelligent analysis, and precise matching. This improves resource utilization efficiency and strengthens grain production capacity. This finding further responds to the existing literature arguing that digital technologies can improve agricultural resource allocation and production efficiency. The difference is that this study not only verifies the overall effect of AI on food supply capacity, but also systematically examines agricultural production efficiency and agricultural resource allocation efficiency as mediating mechanisms, thereby revealing the internal logic through which AI affects food supply capacity.

The heterogeneity analysis further shows that the positive effect of AI on food supply capacity is more pronounced in central China, major grain-producing areas, high-precipitation regions, and regions with more advanced digital infrastructure. This result indicates that AI-enabled improvements in food supply capacity do not occur unconditionally; rather, their actual effects are jointly shaped by regional agricultural foundations, grain production functions, natural climatic conditions, and digital infrastructure levels. Compared with existing studies that mostly discuss the relationship between the digital economy and food security at the national or provincial level, this study uses city-level data to identify the spatial differences.

### 6.2 Policy recommendations

First, efforts should be accelerated to promote the deep integration of AI technologies with agricultural production in order to improve the intelligence level of grain production. Governments should actively promote AI application scenarios throughout the entire grain production process, with particular emphasis on technologies such as intelligent agricultural machinery, remote sensing monitoring, pest and disease identification, precision fertilization, intelligent irrigation, meteorological early-warning systems, and post-harvest grain management. Especially in major grain-producing areas, AI technologies should be embedded into key stages including cultivation, planting, management, harvesting, and storage, thereby improving grain production efficiency and agricultural risk resistance, and facilitating the transformation of grain production from traditional factor-driven growth toward intelligent, precision-oriented, and efficiency-driven development.

Second, rural digital infrastructure should be further improved to provide foundational support for AI-enabled food supply capacity. The effective application of AI technologies depends heavily on network connectivity, data collection, computing power support, and digital platform coordination. Therefore, greater efforts should be made to strengthen rural broadband networks, agricultural Internet of Things (IoT) systems, agricultural big data platforms, intelligent sensing devices, and remote sensing monitoring systems, while narrowing regional disparities in digital infrastructure. For regions with relatively weak digital infrastructure, fiscal investment and policy support should be increased to improve agricultural data acquisition capacity and the coverage of intelligent agricultural services, thereby preventing the emergence of a new “digital divide” resulting from uneven AI adoption.

Third, AI technology applications should be promoted in accordance with local conditions in order to enhance policy precision. The heterogeneity analysis indicates that the impact of AI on food supply capacity differs substantially across regions. Therefore, in central China and major grain-producing areas, policy support should focus on AI applications in grain production, agricultural machinery scheduling, farmland management, and agricultural socialized services. In high-precipitation regions, greater emphasis should be placed on flood monitoring, pest and disease warning systems, and intelligent farmland drainage construction. In low-precipitation regions, policy priorities should include intelligent irrigation systems, water-saving agriculture, and drought monitoring technologies. Through differentiated policy arrangements, the compatibility between AI technologies and regional agricultural production conditions can be significantly improved.

Fourth, mechanisms for improving agricultural production efficiency and agricultural resource allocation efficiency should be strengthened. The mechanism analysis in this study shows that both agricultural production efficiency and resource allocation efficiency are important transmission channels through which AI affects food supply capacity. Therefore, agricultural data-sharing platforms should be accelerated to facilitate the precise allocation of agricultural production factors such as land, labor, capital, machinery, fertilizers, and electricity, thereby reducing resource waste and redundant investment. At the same time, agricultural technology training and the cultivation of new professional farmers should be strengthened to improve the capability of agricultural producers to adopt and utilize AI technologies, ensuring that AI can truly be transformed into practical productive forces that increase grain yield, stabilize production, and improve agricultural quality.

Fifth, a long-term institutional mechanism for AI-enabled food security should be established. Although AI exerts persistent positive effects on food security, its marginal effects may gradually weaken over time. Therefore, continuous efforts should be devoted to promoting AI research and development, technological transformation, and application diffusion in agriculture, while establishing a collaborative innovation system involving governments, research institutions, agricultural enterprises, and farmers. Meanwhile, agricultural data governance systems, technical standards, privacy protection mechanisms, and risk supervision frameworks should be further improved to prevent issues such as data fragmentation, technological monopolies, and regional imbalances in AI applications, thereby ensuring that AI can play a long-term, stable, and sustainable role in safeguarding national food security.

Sixth, while avoiding the emergence of a new rural “digital divide,” policymakers should also pay attention to knowledge gaps and differences in technical capacity among agricultural stakeholders. Governments should strengthen digital skills training for farmers, cooperatives, agricultural enterprises, grassroots agricultural extension workers, and local administrators, thereby improving their understanding and use of AI technologies, agricultural data platforms, smart equipment, and algorithmic decision-making tools. At the same time, agricultural extension systems should be improved so that AI technologies can enter agricultural production scenarios in a lower-cost, more understandable, and more locally adaptable manner.

In addition, consumer communication and information disclosure regarding AI-enabled food applications should be strengthened. For foods involving AI-assisted production, processing, traceability, and recommendation, more transparent mechanisms for quality certification, risk assessment, and information disclosure should be established to enhance consumer understanding of and trust in AI-related foods. Only when infrastructure construction, stakeholder capacity building, and consumer acceptance are advanced together can AI technologies be more effectively transformed into improvements in food supply capacity and broader food security.

## 7 Limitations and future research

### 7.1 Limitations

This study still has several limitations. First, food security is a comprehensive concept that includes multiple dimensions, such as availability, access, utilization, and stability. Due to limitations in the availability of continuous and comparable city-level data, this study uses per capita grain output as a proxy for food supply capacity. This indicator mainly captures the availability dimension of food security, but does not cover residents’ food access, nutritional utilization, or the stability of food supply. Therefore, the findings of this study should be interpreted as evidence of the impact of AI on food supply capacity, rather than as a comprehensive assessment of the full food security system. Future research could further incorporate indicators such as food prices, household income, nutritional consumption structure, grain reserve capacity, market volatility, and climate shocks to construct a multidimensional food security index, thereby providing a more comprehensive evaluation of the overall impact of AI on food security.

Second, the measurement of the AI development index still has certain limitations. The AI development index constructed in this study mainly reflects the overall level of urban AI development, rather than directly measuring the application level of agricultural AI. In particular, indicators such as graphics card imports and Internet penetration can reflect the computing-power foundation and digital connectivity conditions required for urban AI development, but they cannot accurately distinguish whether these technological resources are actually applied to agricultural production. Therefore, the findings of this study mainly reflect the impact of overall urban AI development on food supply capacity. Future research could further incorporate data on agricultural AI firms, smart agriculture patents, smart agricultural machinery, agricultural Internet of Things devices, remote sensing monitoring, and digital farmland construction to develop a more agriculture-specific AI application index, thereby improving the relevance and explanatory power of the measurement.

Third, the mediation analysis in this study also has certain limitations. Although the mediation models include the same control variables as the baseline model and control for city fixed effects and year fixed effects, reverse causality may still exist among agricultural production efficiency, agricultural resource allocation efficiency, and food supply capacity. For example, cities with stronger food supply capacity may have better agricultural foundation conditions and stronger fiscal capacity, which may make it easier for them to promote AI applications and improve agricultural production efficiency. In addition, unobservable factors such as local agricultural governance capacity, agricultural socialized service systems, and the implementation strength of agricultural policies may simultaneously affect AI development, mediating variables, and food supply capacity. Therefore, the mediation results in this study are mainly used to illustrate possible transmission mechanisms through which AI affects food supply capacity, but they cannot fully exclude the influence of reverse mediation or omitted confounding factors. Future research could further use micro-level farmer data, agricultural firm data, or quasi-natural experimental methods to achieve more rigorous causal identification of the mediating mechanisms.

Fourth, this study may still be subject to omitted-variable limitations. Food supply capacity is influenced not only by AI development, but also by factors such as agricultural R&D investment, climate and weather changes, land-use policies, agricultural subsidies, and urban–rural population migration. Although this study includes control variables such as fiscal expenditure, agricultural employment, value added of the primary industry, regional GDP, agricultural machinery input, and fertilizer use, and further controls for city fixed effects and year fixed effects, these controls can only partially mitigate omitted-variable concerns. Due to limitations in the availability of continuous and comparable city-level data, this study cannot fully control for all potential influencing factors. In particular, variables such as agricultural R&D expenditure, specific agricultural subsidy policies, the intensity of land-use policies, and urban–rural migration flows may exhibit strong regional differences and temporal variation. Future research could further combine county-level data, remote sensing land-use data, agricultural policy text data, and population mobility data to more systematically identify the effects of these factors on food supply capacity and further examine the robustness of the role of AI.

### 7.2 Future research

It should be noted that the impact of AI on food supply capacity is not exclusively positive. The empirical results of this study show that AI development generally helps improve urban food supply capacity. However, during the transition toward agricultural intelligence, it remains necessary to pay attention to the potential negative effects associated with AI adoption.

First, AI may lead to agricultural labor displacement. Smart agricultural machinery, automated equipment, and algorithmic decision-making systems can replace some repetitive and low-skilled agricultural labor, thereby improving production efficiency. However, they may also reduce employment opportunities for some agricultural workers, especially older workers, low-skilled workers, and those with limited digital capabilities. Without corresponding skills training and employment transition support, AI adoption may intensify inequality within the agricultural labor force.

Second, AI applications may increase energy consumption and environmental pressure. AI model training, remote sensing image processing, the operation of agricultural data platforms, the use of smart devices, and the construction of data centers all require computing power and electricity. If the relevant energy supply still relies heavily on high-carbon energy sources, AI may generate additional energy consumption and carbon emission pressures while improving agricultural production efficiency. Therefore, the promotion of agricultural AI applications should be combined with green computing power, energy-efficient equipment, and low-carbon digital infrastructure.

Third, AI may widen the digital divide across regions and among different agricultural operators. The effective use of AI technologies depends on network infrastructure, data resources, smart equipment, capital investment, and digital skills. Regions with weak digital infrastructure, small-scale farmers, and groups with limited digital skills may find it difficult to fully benefit from AI technologies. By contrast, large agricultural enterprises, platform enterprises, and operators with stronger capital capacity are more likely to benefit first. This may lead to an uneven distribution of AI-related benefits among agricultural actors.

Finally, AI applications may also lead to agricultural data concentration and platform dependence. Agricultural production data, remote sensing data, agricultural machinery operation data, and market transaction data all have important economic value. If such data become excessively concentrated in the hands of a few platforms or enterprises, farmers and local agricultural actors may lose control over their own data and become increasingly dependent on external platforms and algorithmic systems. Data concentration may also affect the bargaining power and technological autonomy of agricultural producers.

It should be further clarified that although this study takes food supply capacity as the empirical object of analysis, its research logic is closely related to the broader topic of artificial intelligence-enabled agri-food systems and food security. Food supply capacity represents a fundamental dimension of food security, while the impact of artificial intelligence on food security is not limited to the agricultural production stage. At the production stage, AI can improve grain output capacity and production stability through precision agriculture, intelligent agricultural machinery, remote sensing monitoring, pest and disease identification, smart irrigation, and agricultural risk early-warning systems. In the post-production stage, AI may also improve food supply efficiency and system resilience through intelligent storage, logistics route optimization, quality traceability, enhanced supply chain transparency, and market demand forecasting.

Due to the limitation of long-term and continuous city-level data availability, this study focuses on examining the impact of AI development on food supply capacity, while it does not systematically investigate the role of AI in food processing, storage and logistics, distribution, consumption choices, and nutritional utilization. Therefore, the findings of this study mainly apply to the availability dimension of food security. Future research could further incorporate agri-food supply chain data, enterprise logistics data, food loss data, consumer survey data, and nutrition-related data to examine the comprehensive impact of AI on the multidimensional objectives of food security from a complete “farm-to-table” perspective.

Therefore, in the process of promoting AI-enabled improvements in food supply capacity, policymakers should not only focus on technological efficiency and output growth, but also pay attention to the inclusiveness, sustainability, and governance of AI applications. Future policies should strengthen digital skills training and employment transition support for agricultural workers, promote green computing power and low-carbon digital infrastructure, improve rules on agricultural data governance, data sharing, and privacy protection, and reduce the barriers faced by smallholder farmers in using AI technologies through public digital service platforms. In this way, potential negative effects can be reduced while food supply capacity is improved.

The mechanism analysis in this study remains mainly focused on the agricultural production side and does not systematically examine the role of AI in downstream agri-food supply chains. In fact, AI may affect food security through food processing, storage and logistics, distribution, improved supply chain transparency, reduced post-harvest losses, lower food waste, food quality traceability, and personalized nutrition. At the same time, consumers’ trust, perceptions, and acceptance of AI-produced or AI-assisted food may also influence the diffusion and practical effectiveness of AI technologies in agri-food systems. Due to limitations in the availability of long-term continuous city-level data, these variables are not included in the empirical model of this study. Future research could further use agri-food supply chain data, firm-level logistics data, food loss data, consumer survey data, and platform transaction data to examine the comprehensive impact of AI on food security from a full “farm-to-fork” perspective.

This study examines the impact of AI development on food supply capacity mainly from the perspective of city-level digital infrastructure and AI development. However, it does not directly measure the knowledge level, digital skills, technology acceptance capacity of agricultural stakeholders, or consumer trust in AI-related food. In fact, even when digital infrastructure is relatively well developed, the actual effectiveness of AI technologies may still be constrained if farmers and agricultural operators lack the necessary knowledge and skills, or if consumers remain skeptical about AI-produced or AI-assisted food. Future research could combine farmer survey data, agricultural extension data, consumer survey data, and food market data to further examine the roles of knowledge gaps, technology acceptance, and consumer trust in the relationship between AI and food security.

## Data Availability

The original contributions presented in the study are included in the article/supplementary material, further inquiries can be directed to the corresponding author.
